# A Foundation Model Based CT Biomarker for Non‐Invasive Prediction of Response to Neoadjuvant Immunochemotherapy in Non‐Small Cell Lung Cancer

**DOI:** 10.1002/advs.75933

**Published:** 2026-06-02

**Authors:** Yanglan Xu, Shuchang Zhou, Qin Peng, Xiao Bao, Xiaodan Ye, Ahmet Görkem Er, Tong Tong, Mirabela Rusu, Yajia Gu, Mailin Chen, Jing Gong

**Affiliations:** ^1^ Department of Radiology Fudan University Shanghai Cancer Center Shanghai China; ^2^ Department of Oncology Shanghai Medical College Fudan University Shanghai China; ^3^ Department of Radiology Tongji Hospital Tongji Medical College Huazhong University of Science and Technology Wuhan China; ^4^ Department of Radiology Shanghai Pulmonary Hospital Shanghai China; ^5^ Department of Radiology Zhongshan Hospital Fudan University Shanghai China; ^6^ Shanghai Institute of Medical Imaging Shanghai China; ^7^ Department of Cancer Center Zhongshan Hospital Fudan University Shanghai China; ^8^ Department of Radiology Stanford University Stanford California USA; ^9^ Key Laboratory of Carcinogenesis and Translational Research (Ministry of Education/Beijing) Department of Radiology Peking University Cancer Hospital & Institute Beijing China

**Keywords:** deep learning, foundation model, imaging biomarker, neoadjuvant immunochemotherapy, non‐small cell lung cancer

## Abstract

Predicting pathological complete response (pCR) to neoadjuvant immunochemotherapy in non‐small cell lung cancer (NSCLC) is clinically important yet remains challenging. Here, we introduce a foundation model‐derived computed tomography (CT) imaging biomarker established from a multi‐center cohort of 702 patients. Specifically, we developed and validated a non‐invasive baseline CT‐based model for risk stratification of pathological response. To address scanner and protocol heterogeneity, we first built a 3D Vision Mamba‐based CT super‐resolution model trained on 2494 cases for image standardization. We then fine‐tuned a lung cancer‐specific CT foundation model from a pretrained 3D model (VoCo) using 6643 chest CT scans. Finally, we constructed a multi‐task Swin Transformer that jointly performs risk stratification and segments tumors to generate the imaging biomarker. Across five centers, the model achieved consistently strong generalization (AUC: 0.75–0.87) for pCR prediction. Genomic analysis revealed that the biomarker was independent of tumor mutational burden but significantly associated with *TP53* mutations, suggesting an association with a radiogenomic phenotype related to this alteration. Together, these results demonstrate a generalizable and biologically meaningful foundation model‐based biomarker for non‐invasive risk stratification of pathological response in NSCLC.

## Graphical abstract

This study introduces a foundation model‐based biomarker for risk stratification of pathological response in non‐small cell lung cancer. A Vision Mamba super‐resolution model standardizes heterogeneous CT images. A multi‐task Swin Transformer then fine‐tunes a pre‐trained lung foundation model to jointly optimize tumor segmentation and response prediction. Cross‐cohort validation demonstrates its robust generalization and clinical translatability.

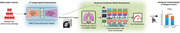

## 1 Introduction

Lung cancer remains a leading cause of global cancer mortality, with non‐small cell lung cancer (NSCLC) comprising 85% of cases [1, 2]. Immunotherapy has emerged as a cornerstone of NSCLC management and is reshaping the therapeutic landscape through neoadjuvant immunochemotherapy (NCI) [3, 4, 5]. Notably, the CheckMate‐816 trial demonstrated that NCI significantly improves pathological complete response (pCR) rates compared to chemotherapy alone, without increasing adverse event rates [6]. Studies have shown that patients treated with immunotherapy may achieve good long‐term survival outcomes without requiring surgery [7, 8, 9], which underscores the urgent and unmet need to identify NSCLC patients who might positively respond to this therapeutic strategy. However, robust predictive biomarkers for NCI efficacy are lacking. Although programmed death‐ligand 1 (PD‐L1) expression has been identified as a potential predictive marker, its role in resectable NSCLC remains controversial, and its assessment necessitates an invasive biopsy [6, 10]. Therefore, the development of a robust, non‐invasive imaging biomarker to predict pCR following NCI in NSCLC is important.

Computed tomography (CT) imaging is a standard, cost‐effective tool for monitoring tumor response to treatment in lung cancer [11]. However, a substantial discrepancy exists between radiological and pathological assessments when evaluating the response to NCI [12]. It is therefore necessary to refine methodologies to bridge this gap between radiological and pathological evaluations in the context of NCI. Artificial intelligence (AI) offers a solution by extracting subtle features from CT images, which can enhance diagnostic and prognostic accuracy of lung cancer [13, 14, 15]. Foundation models represent a new frontier in medical AI research and development [16, 17]. These models are pre‐trained on massive, diverse datasets and can be adapted to numerous downstream tasks with minimal or no additional training [18, 19, 20]. This presents a significant advantage over conventional approaches, as traditional deep learning feature extraction methods are often based on models trained on natural images or limited datasets, necessitating a new model for each task and limiting their applicability in medical research [14, 21].

**FIGURE 1:**
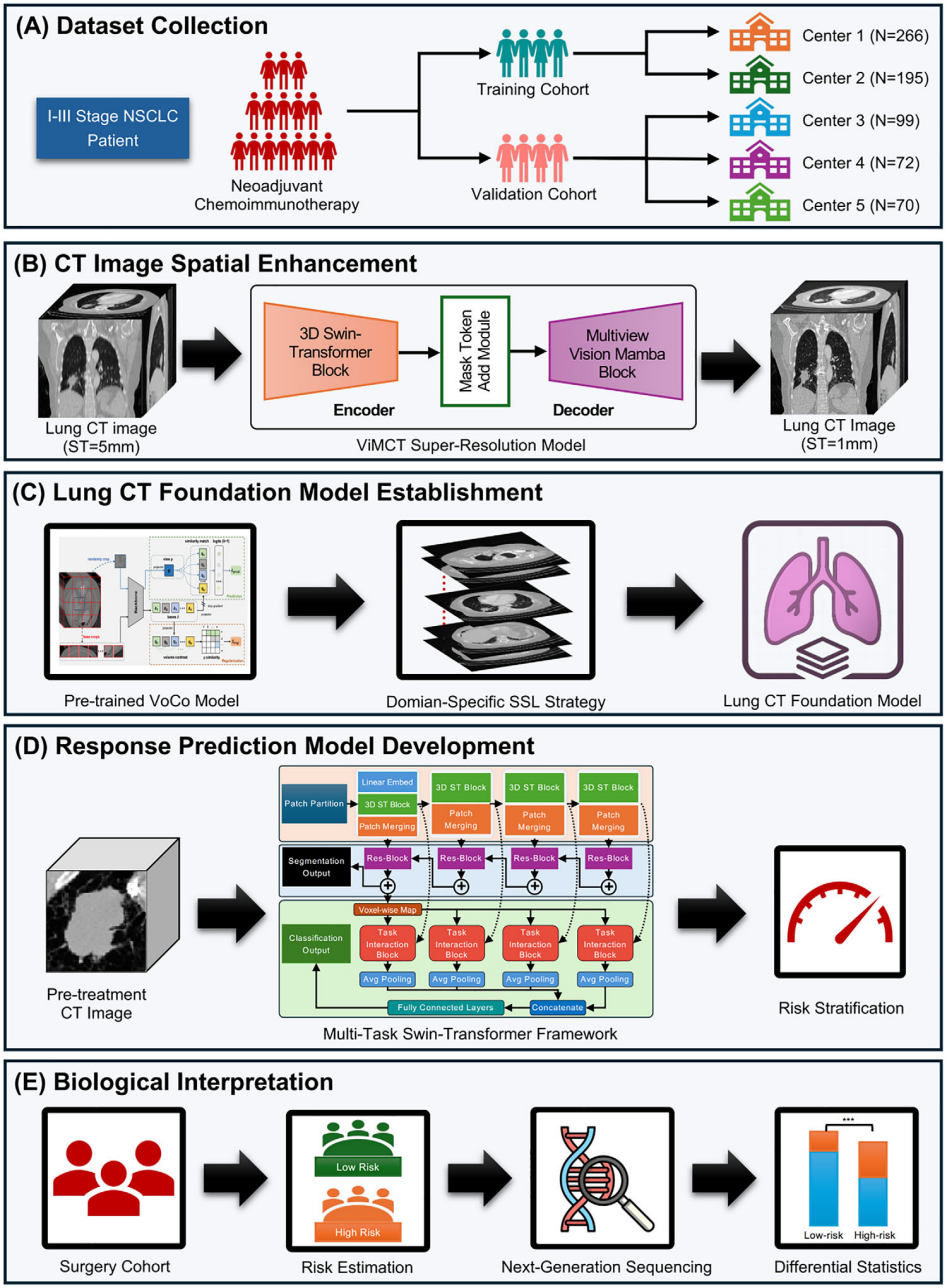
Overview of the multi‐stage deep learning framework and study design. (A) Dataset Collection: The study cohort comprises 702 NSCLC patients treated with NCI from five centers, divided into a primary Training set (n = 461) and three External Validation sets (n = 241 total). (B) CT Image Spatial Enhancement: The ViMCT model is employed to convert heterogeneous thick‐slice CTs (ST = 5 mm) into standardized synthetic thin‐slice CTs (ST = 1 mm). (C) Lung CT Foundation Model Establishment: The VoCo model is pre‐trained using a Domain‐Specific SSL strategy on our lung CT data to establish a robust Lung CT Foundation Model. (D) Response Prediction Model Development: The MTST framework is developed upon the foundation model. MTST synergistically performs tumor segmentation and pCR classification, using the segmentation output (Voxel‐wise Map) to spatially guide the classification for improved risk stratification. (E) Biological Interpretation: The model's risk stratification results from an independent surgery cohort are correlated with NGS data. This radiogenomic approach provides biological interpretation for the model's prediction of pathological complete response. Abbreviations: NSCLC, non‐small cell lung cancer; NCI, neoadjuvant immunochemotherapy; ST, slice thickness; ViMCT, Vision Mamba CT super‐resolution network; VoCo, volume contrast; SSL, self‐supervised learning; MTST, multi‐task Swin‐Transformer; pCR, pathological complete response; NGS, next‐generation sequencing.

In this study, to develop CT biomarker for NCI response of NSCLC, we utilized the VoCo foundation model to build and evaluate a domain‐specific model on a dataset with 702 cases from five centers. The VoCo model was pre‐trained as a general medical foundation model on a large‐scale, multi‐modal dataset comprising CT and MR images [22]. We first developed a Vision Mamba CT super‐resolution network (ViMCT) using 2494 paired CT scans to standardize images. Then, we fine‐tuned the VoCo model on our lung CT dataset (n = 6643) using a domain‐specific self‐supervised learning (SSL) strategy. This process enabled the model to specifically learn pulmonary imaging features, yielding a Lung CT Foundation Model. Using this lung foundation model as a starting point, we fine‐tuned it into a Multi‐Task Swin‐Transformer (MTST) framework that synergistically performs both tumor segmentation and pCR classification. Subsequently, we performed supervised training on this multi‐task framework using labeled pre‐treatment CT images to achieve precise risk stratification. We provide an overview of our entire pipeline in Figure 1. A detailed description of our methodology is provided in the Methods.

## 2 Results

### 2.1 Baseline Clinical Characteristics

**FIGURE 2:**
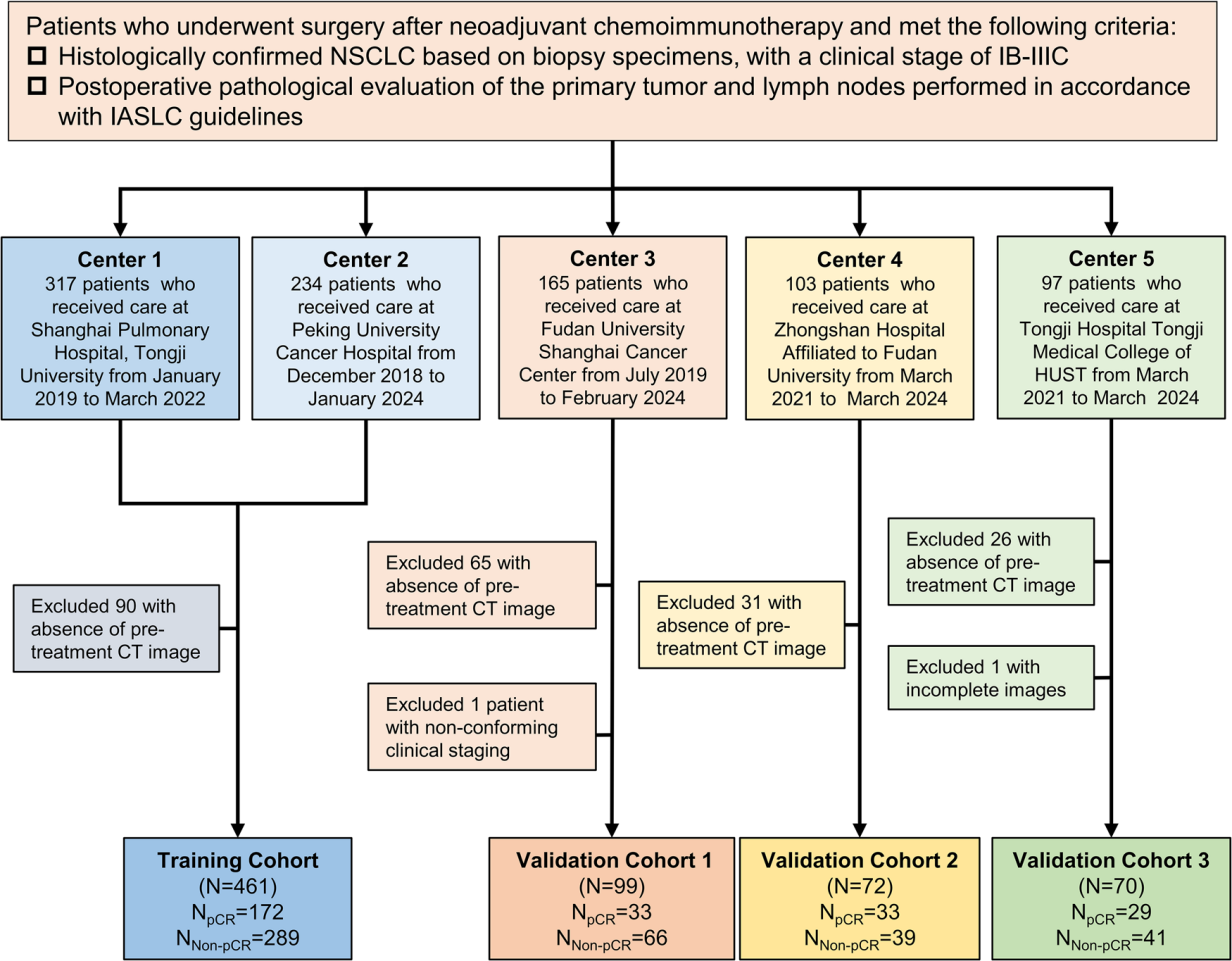
Flowchart of patient enrollment and cohort allocation. This flow diagram illustrates the cohort allocation for the multi‐center study evaluating NCI in resectable NSCLC. The study initially recruited 916 patients (2018–2024). Ultimately, 702 eligible patients were allocated into the following groups: n = 461 patients formed the Training Cohort, while the remaining n = 241 patients (from three external centers) served as the Validation Cohorts. Abbreviations: NCI, neoadjuvant immunochemotherapy; NSCLC, non‐small cell lung cancer; pCR, pathological complete response.

Data from 916 patients who received NCI prior to surgery were initially collected for this retrospective study across five centers between December 2018 and March 2024, according to the inclusion criteria. Of these, 317 patients were retrospectively recruited from Shanghai Pulmonary Hospital, Tongji University (Center 1); 234 from Peking University Cancer Hospital (Center 2); 165 from Fudan University Shanghai Cancer Center (Center 3); 103 from Zhongshan Hospital Affiliated to Fudan University (Center 4); and 97 from Tongji Hospital, Tongji Medical College of Huazhong University of Science and Technology (Center 5). After screening, 212 patients were excluded due to missing CT scans, one was excluded for non‐conforming clinical staging, and one was excluded due to incomplete images. A flowchart of the patient enrollment process is shown in Figure 2.

Ultimately, this study enrolled 702 eligible patients. Patients from Center 1 (n = 266) and Center 2 (n = 195) constituted the training cohort (n = 461). Patients from Center 3 (n = 99), Center 4 (n = 72), and Center 5 (n = 70), all enrolled in the same prospective multicenter observational study (NCT04766515), were used as three external validation cohorts. The proportion of patients achieving pCR was 37.3% in the training cohort, and 33.3%, 45.8%, and 41.4% in the three external validation cohorts, respectively. The clinical characteristics of patients in the training and three validation cohorts are summarized in Table 1. Significant differences (p < 0.05) were observed between the pCR and non‐pCR groups regarding sex, smoking history, histological subtype, N stage, and clinical stage.

**TABLE 1:** Clinical characteristics of the patients in training and three validation cohorts.

Characteristic	Training Cohort		*p* Value	Validation Cohort 1		*p* Value	Validation Cohort 2		*p* Value	Validation Cohort 3		*p* Value
	Non‐pCR (N = 289)	pCR (N = 172)		Non‐pCR (N = 66)	pCR (N = 33)		Non‐pCR (N = 39)	pCR (N = 33)		Non‐pCR (N = 41)	pCR (N = 29) (N = 24)	
Sex			0.0026			0.8711			0.5717			1.0000
Male	251 (86.9%)	165 (95.9%)		62 (93.9%)	32 (97.0%)		34 (87.2%)	31 (93.9%)		38 (92.7%)	27 (93.1%)	
Female	38 (13.1%)	7 (4.1%)		4 (6.1%)	1 (3.0%)		5 (12.8%)	2 (6.1%)		3 (7.3%)	2 (6.9%)	
Age (years)	61.6 ± 8.7	62.6 ± 7.2	0.1951	62.9 ± 7.6	61.3 ± 9.1	0.4002	63.2 ± 7.5	63.0 ± 6.5	0.9295	60.9 ± 5.9	63.1 ± 6.0	0.1421
Smoking History			0.0002			0.7440			1.0000			0.9157
Yes	204 (70.6%)	148 (86.0%)		57 (86.4%)	30 (90.9%)		27 (69.2%)	23 (69.7%)		28 (68.3%)	21 (72.4%)	
No	85 (29.4%)	24 (14.0%)		9 (13.6%)	3 (9.1%)		12 (30.8%)	10 (30.3%)		13 (31.7%)	8 (27.6%)	
Pathology Subtype			0.0002			0.3584			0.0182			0.0097
SCC	175 (60.6%)	127 (73.8%)		54 (81.8%)	24 (72.7%)		23 (59.0%)	29 (87.9%)		21 (51.2%)	25 (86.2%)	
ADC	85 (29.4%)	22 (12.8%)		9 (13.6%)	5 (14.7%)		13 (33.3%)	4 (12.1%)		14 (34.1%)	3 (10.3%)	
Other	29 (10.0%)	23 (13.4%)		3 (4.5%)	4 (12.1%)		3 (7.7%)	0 (0.0%)		6 (14.6%)	1 (3.4%)	
T			0.2278			0.7764			0.2819			0.1039
T1	31 (18.0%)	37 (12.8%)		8 (12.1%)	4 (12.1%)		9 (23.1%)	5 (15.2%)		17 (41.5%)	6 (20.7%)	
T2	96 (33.2%)	64 (37.2%)		24 (36.4%)	12 (36.4%)		13 (33.3%)	18 (54.5%)		13 (31.7%)	7 (24.1%)	
T3	81 (28.0%)	41 (23.8%)		21 (31.8%)	13 (39.4%)		11 (28.2%)	5 (15.2%)		6 (14.6%)	8 (27.6%)	
T4	75 (26.0%)	36 (20.9%)		13 (19.7%)	4 (12.1%)		6 (15.4%)	5 (15.2%)		5 (12.2%)	8 (27.6%)	
N			0.0943			0.2984			0.5971			0.0093
N0	50 (17.3%)	20 (11.6%)		11 (16.7%)	4 (12.1%)		5 (12.8%)	6 (18.2%)		17 (41.5%)	2 (6.9%)	
N1	69 (23.9%)	55 (32.0%)		14 (21.2%)	13 (39.4%)		9 (23.1%)	4 (12.1%)		11 (37.9%)	10 (24.4%)	
N2	162 (56.1%)	89 (51.7%)		28 (42.4%)	11 (33.3%)		23 (59.0%)	22 (66.7%)		12 (29.3%)	11 (37.9%)	
N3	8 (2.8%)	8 (4.7%)		13 (19.7%)	5 (15.2%)		2 (5.1%)	1 (3.0%)		2 (4.9%)	5 (17.2%)	
TNM Stage			0.2640			0.5114			0.6972			0.0258
IB	6 (2.1%)	4 (2.3%)		3 (4.5%)	0 (0.0%)		3 (7.7%)	3 (9.1%)		12 (29.3%)	1 (3.4%)	
IIA	6 (2.1%)	3 (1.7%)		6 (9.1%)	3 (9.1%)		0 (0.0%)	1 (3.0%)		4 (9.8%)	2 (6.9%)	
IIB	39 (13.5%)	38 (22.1%)		10 (15.2%)	7 (21.2%)		6 (15.4%)	3 (9.1%)		6 (14.6%)	4 (13.8%)	
IIIA	162 (56.1%)	85 (49.4%)		12 (18.2%)	10 (30.3%)		20 (51.3%)	16 (48.5%)		15 (36.6%)	11 (37.9%)	
IIIB	73 (25.3%)	39 (22.7%)		27 (40.9%)	10 (30.3%)		10 (25.6%)	9 (27.3%)		2 (4.9%)	7 (24.1%)	
IIIC	3 (1.0%)	3 (1.7%)		8 (12.1%)	3 (9.1%)		0 (0.0%)	1 (3.0%)		2 (4.9%)	4 (13.8%)	

### 2.2 Synthetic Thin‐Slice CT Generation and Quantitative Image Quality Evaluation

The multicenter NCI cohort exhibited considerable heterogeneity in CT acquisition and reconstruction protocols, as summarized in Table S1. A considerable proportion of CT scans were reconstructed with relatively thick slices, with slice thicknesses of up to 5 mm. we developed a ViMCT (as shown in Figure 1A) to generate thin‐slice CT and standardize the dataset. To train this model, we collected 2494 matched pairs of 5 mm thick‐slice and 1 mm thin‐slice chest CT images from Fudan University Shanghai Cancer Center. The ViMCT encoder utilized a 3D Swin‐Transformer module to capture both local and global image features, while the decoder employed a multiview vision Mamba block, which combines global modeling capabilities with high computational efficiency. For performance evaluation, we introduced two methods for comparison: a traditional 3D Trilinear Interpolation (TIP) method, and the Transformer Volumetric Super‐Resolution Network (TVSR) [23]. We processed the original thick‐slice CTs from each test set using these three methods.

**FIGURE 3:**
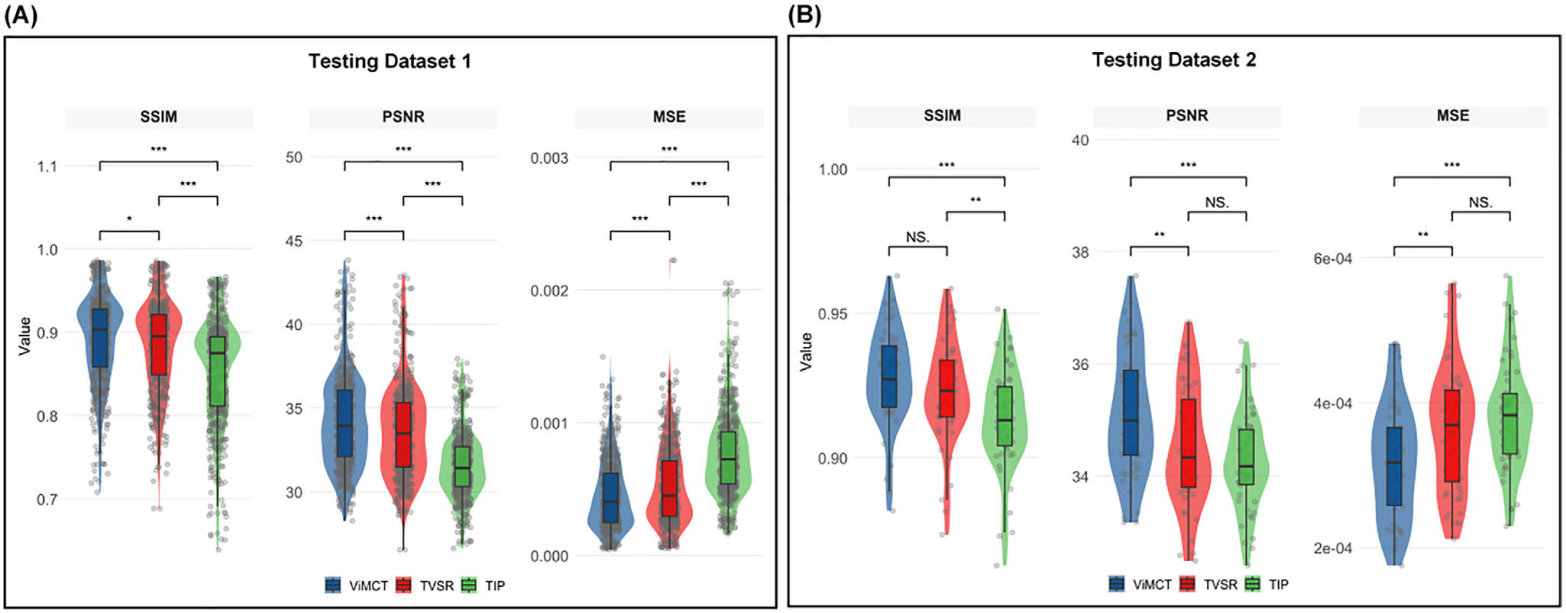
Quantitative evaluation of the ViMCT synthetic thin‐slice CT model. Violin plots for (A) Testing Dataset 1 and (B) Testing Dataset 2 compare the performance of the ViMCT model against the TVSR model and TIP across three key quantitative metrics: SSIM, PSNR, and MSE.The ViMCT model demonstrated superior super‐resolution performance across all key metrics and both independent test sets, outperforming the traditional TIP method and the previous state‐of‐the‐art TVSR model. Abbreviations: SSIM, structural similarity index measure; PSNR, peak signal‐to‐noise ratio; MSE, mean squared error; TIP, trilinear interpolation; NS., not statistically significant.

The violin plots in Figure 3 illustrate the image quality comparison of the three methods based on quantitative metrics, including Peak Signal‐to‐Noise Ratio (PSNR), Structural Similarity Index Measure (SSIM), and Mean Squared Error (MSE). In Testing Dataset 1 (n = 256, Fudan University Shanghai Cancer Center) and Testing Dataset 2 (n = 50, RPLHR‐CT‐tiny dataset, https://github.com/smilenaxx/CTHNet‐CT‐Slice‐Synthesis), the ViMCT model outperformed the traditional TIP method (p < 0.01) and the previous TVSR model (p < 0.05).

### 2.3 Quantitative Performance Evaluation of MTST Model on Segmentation and Classification Tasks

To achieve accurate pCR prediction, we first leveraged our lung CT data to fine‐tune VoCo with 6,643 CT scans to develop a specialized lung foundation model (Figure 1B). Then, we developed the MTST model to predict the pCR of NSCLC patients treated with NCI (Figure 1C; detailed architecture in Figure S1). Subsequently, we performed supervised training to generate the final risk stratification (Figure 1D). To evaluate segmentation performance, we compared MTST against single task Swin‐UNEt TRansformers (Swin‐UNETR) [24], a state‐of‐the‐art hybrid architecture.

**FIGURE 4:**
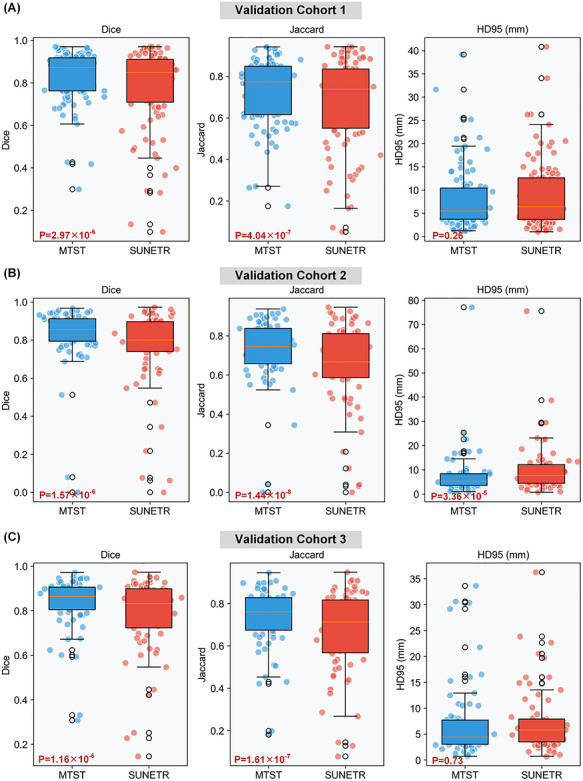
Comparison of model segmentation performance. Box plots illustrate the quantitative segmentation performance of the MTST model versus the SUNETR across three external validation cohorts. Metrics include the Dice coefficient, Jaccard coefficient, and HD95, measuring boundary accuracy. Results show that MTST achieved significantly higher Dice and Jaccard coefficients in all cohorts, with superior HD95 performance in one cohort, confirming MTST's advantage in tumor segmentation. Abbreviations: HD95, 95% hausdorff distance; NS., not statistically significant.

The boxplots in Figure 4 show the quantitative evaluation of segmentation performance, including the Dice coefficient, Jaccard coefficient, and 95% Hausdorff Distance (HD95). MTST demonstrated significantly higher Dice and Jaccard coefficients than UNETR in Validation Cohort 1 (p = 2.97 × 10^−6^ and p = 4.04 × 10^−7^), Cohort 2 (p = 1.57 × 10^−6^ and p = 1.44 × 10^−8^), and Cohort 3 (p = 1.16 × 10^−6^ and p = 1.61 × 10^−7^). For the HD95 metric, MTST was significantly lower in Cohort 2 (p = 3.38 × 10^−5^), with no significant difference in Cohorts 1 and 3 (p = 0.26 and p = 0.73, respectively). In summary, MTST significantly outperformed Swin‐UNETR on the Dice and Jaccard coefficients, while achieving comparable or better performance on the HD95 metric across all cohorts.

**FIGURE 5:**
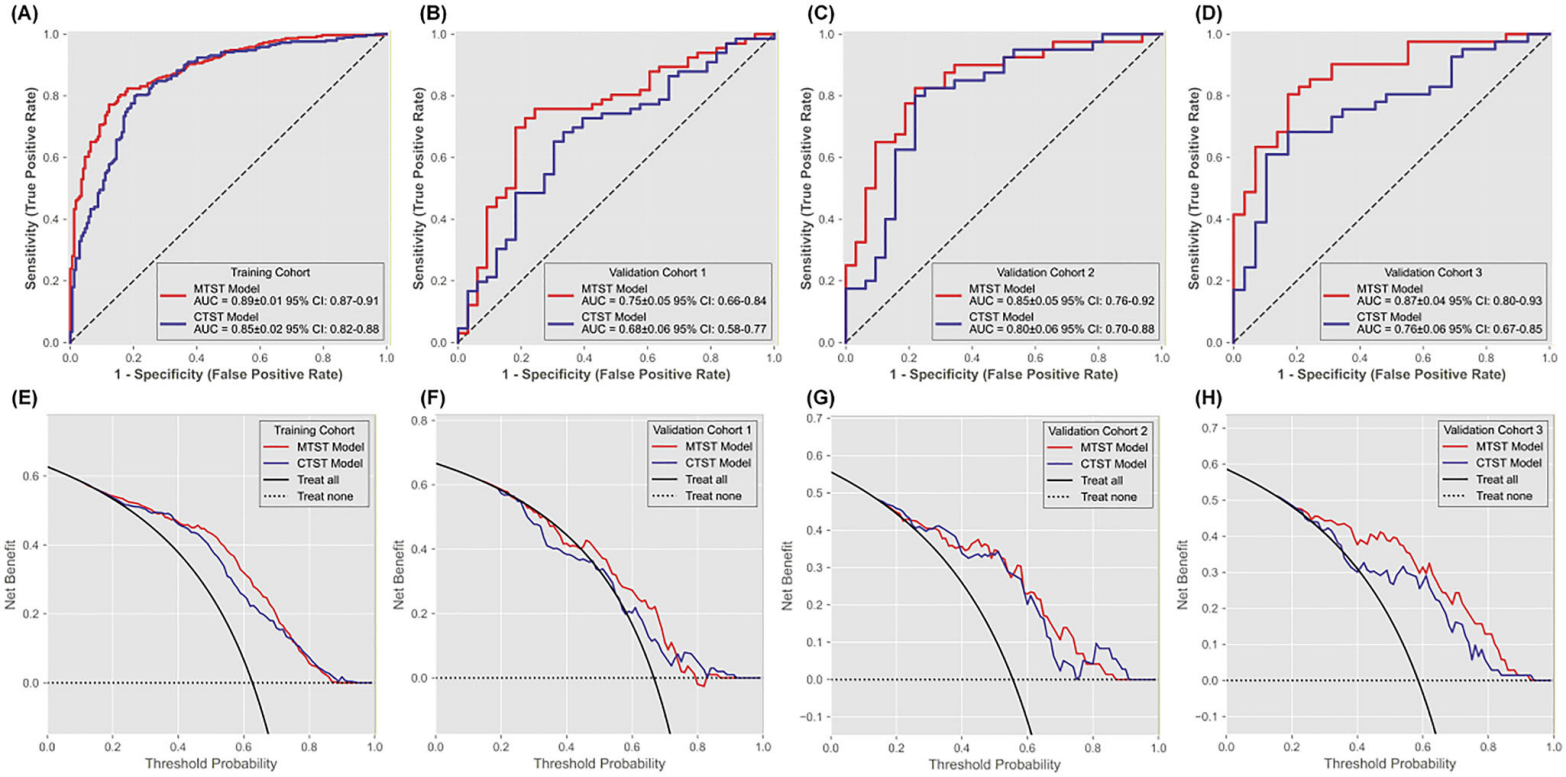
Comparison of model classification performance. Panels (A) to (D) display the ROC curve analysis, comparing the discriminative performance of the MTST model (blue) and CTST model (red) across the training and three external validation cohorts. MTST consistently achieved a higher AUC in all cohorts. Panels (E) to (H) display the DCA, comparing the net benefit of MTST and CTST across different decision thresholds. MTST consistently showed a higher Net Benefit than CTST across a wide range of threshold probabilities, confirming MTST's superior clinical utility in therapeutic decision‐making. Abbreviations: AUC, area under the curve; DCA, decision curve analysis; pCR, pathological complete response; MTST, multi‐task Swin‐Transformer; CTST, single classification‐task Swin‐Transformer.

Figure 5 compares the performance of the MTST model with the single Classification‐Task Swin‐Transformer (CTST) model on the pCR classification task. ROC curve analysis (Figure 5A–D) showed that the AUCs for MTST in the training cohort, Validation Cohort 1, 2, and 3 were 0.89 ± 0.01 (95% CI: 0.87‐0.91), 0.75 ± 0.05 (95% CI: 0.66‐0.84), 0.85 ± 0.05 (95% CI: 0.76‐0.92), and 0.87 ± 0.04 (95% CI:0.80‐0.93), respectively. All were higher than those of the CTST (corresponding AUCs: 0.82, 0.68, 0.82, and 0.76), indicating MTST's superior classification accuracy. The Decision Curve Analysis (DCA) in Figure 5E–H demonstrated that MTST achieved a higher net benefit than CTST in all cohorts. The calibration curves (Figure S3) indicated the MTST model was well‐calibrated across the training cohort and most validation cohorts. In contrast, the CTST model showed general volatility and weaker overall calibration performance than MTST. The confusion matrix for the MTST model is shown in Figure S2. Table S2 details the other key evaluation metrics, including accuracy (ACC), sensitivity (SEN), specificity (SPE), positive predictive value (PPV), and negative predictive value (NPV). The model maintained robust performance across the three validation cohorts, with ACCs ranging from 0.727 to 0.800, and consistently demonstrated high SPE (0.821–0.882) and high PPV (0.781–0.828) across all validation cohorts. We also compared the MTST model with representative baseline approaches on validation cohort 1. As shown in Table S3, the MTST framework yielded superior predictive performance over the established traditional radiomics and habitat imaging models. Meanwhile, Table S4 provides a systematic summary of recent related studies, comparing our framework with previous works in terms of cohort size, model architecture, imaging input, and reported validation performance. Furthermore, a multimodal fusion model integrating clinicopathological features demonstrated no significant improvement over the MTST model (Figure S4).

### 2.4 Workflow Overview and Representative Case Analyses

**FIGURE 6:**
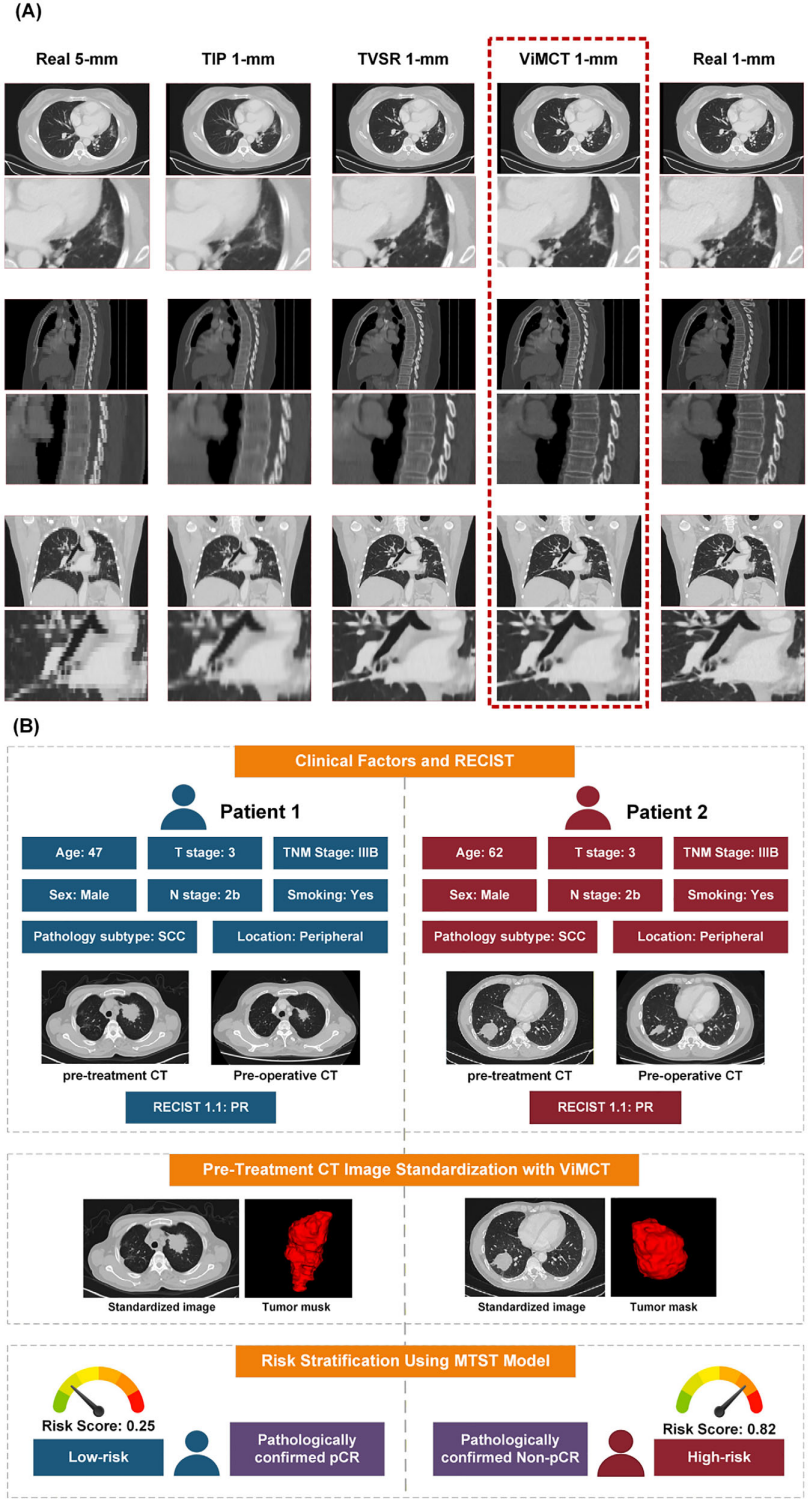
Workflow illustration and representative qualitative results. (A) Visual quality assessment of the ViMCT model. Comparison for a representative patient from the Fudan University Shanghai Cancer Center, showing the original 5 mm thick‐slice CT, the real 1 mm thin‐slice CT, and the synthetic results from TIP, the TVSR model, and the ViMCT model. Axial and coronal views are displayed in the lung window, and sagittal views are shown in the bone window. (B) Clinical applicability of the MTST model. Application of the framework to two patients with similar baseline clinical characteristics. Conventional RECIST 1.1 criteria classified both cases as Partial Response. In contrast, the MTST model differentiated the patient who achieved pCR (Patient 1: low‐risk prediction, score 0.25) from the non‐pCR patient (Patient 2: high‐risk prediction, score 0.82) using only pretreatment CT imaging. Abbreviations: TIP, trilinear Interpolation; pCR, pathological complete response; PR, partial response.

To demonstrate both the visual fidelity of ViMCT and the clinical applicability of MTST, Figure 6 presents representative case studies. Figure 6A provides a multi‐planar comparison (axial, sagittal, coronal) of ViMCT against other SR methods. The ViMCT‐generated 1 mm images most closely approximate the structural detail of the real 1 mm thin‐slice CT, confirming its superior reconstruction quality. Figure 6B illustrates the application of framework to two patients with nearly identical baseline clinical characteristics, including pre‐treatment clinical staging (T, N, and TNM stage), smoking status, age, pathological type, tumor location, and tumor volume. The MTST model assigned a low‐risk score of 0.25 to Patient 1 and a high‐risk score of 0.82 to Patient 2. Pathology confirmed that Patient 1 achieved pCR, whereas Patient 2 did not. Notably, both patients were classified as partial response (PR) by RECIST 1.1 criteria [25], highlighting RECIST's limitations in distinguishing biologically distinct outcomes. These examples demonstrate that our MTST model can accurately predict pathological response from baseline CT images alone, revealing radiological phenotypes linked to treatment outcome that remain undetected by conventional RECIST assessment. To improve interpretability, we added representative segmentation maps and Grad‐CAM++ visualizations (Figure S5). These analyses suggest that the model attends to different regions in non‐pCR and pCR cases: in non‐pCR cases, attention is more concentrated in the tumor center and internally heterogeneous regions, whereas in pCR cases, attention extends more toward the tumor margin and the tumor‐lung interface.

### 2.5 Biological Interpretability Analysis

**FIGURE 7:**
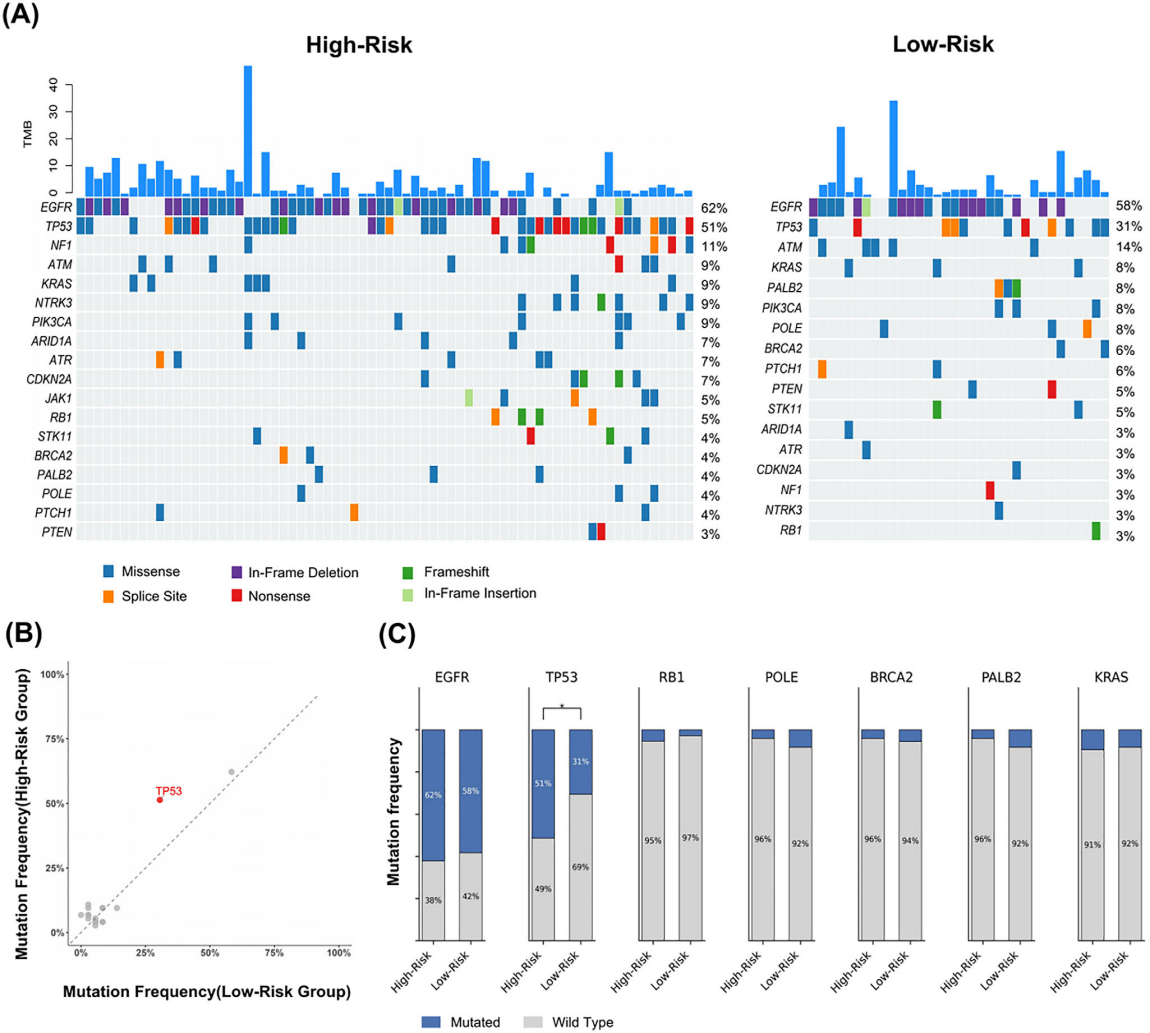
Association analysis between MTST model risk stratification and the tumor genomic landscape. This figure illustrates the genomic mutation profile in the MTST model's predicted high‐risk (non‐pCR) and low‐risk (pCR) cohorts, exploring the biological basis underlying the predictions. (A) TMB and Waterfall Plot: The TMB distribution plot above shows no significant difference in tumor mutation burden between the high‐risk and low‐risk groups (p = 0.56655). The waterfall plot below details the mutation types and distribution of driver genes with a mutation frequency greater than 3%. (B) Mutation Frequency Correlation Scatter Plot: Compares the gene mutation frequency between the high‐ and low‐risk groups. Red dots indicate genes with a significant difference (p < 0.05). *TP53* was the only driver gene showing a significant difference between the two groups (p < 0.05), suggesting a close association between *TP53* mutation and the model's predicted high‐risk phenotype. (C) Key Gene Mutation Status Stacked Bar Plot: Details the mutation proportions of key genes, such as *TP53*, *EGFR*, *KRAS*, and *RB1*, in the high‐ and low‐risk groups. The *TP53* mutation rate in the high‐risk group (51.4%) was significantly higher than in the low‐risk group (30.6%, p < 0.05). In contrast, the mutation proportions for genes like *EGFR*, *KRAS*, and *RB1* did not show a statistically significant difference between the two groups. Abbreviations: TMB, tumor mutation burden; pCR, pathological complete response.

To elucidate the biologic mechanisms behind the CT imaging model, we retrospectively collected 520‐gene NGS panel data from 110 patients at Fudan University Shanghai Cancer Center who underwent direct surgical resection for lung cancer. The 110 patients were divided into a high‐risk group (n = 74, predicted as non‐pCR) and a low‐risk group (n = 36, predicted as pCR) by using MTST model. Figure 7 A illustrates the distribution overview of driver gene mutation with a mutation frequency >3% in both the high‐ and low‐risk groups. It revealed no significant difference in tumor mutation burden (TMB) between the two groups (median: 2.99 vs. 3.00 muts/Mb, p = 0.56655). Figure 7B presents the correlation of mutation frequencies between these two groups. It revealed that *TP53* mutations were significantly more frequent in the high‐risk group than in the low‐risk group (P<0.05), suggesting a strong association between *TP53* mutation and the high‐risk phenotype. Figure 7C further details the mutation proportions of key genes, showing that *TP53* mutation rate in the high‐risk group was 51.4%, significantly higher than the 30.6% observed in the low‐risk group (p < 0.05). In contrast, although other genes such as *EGFR*, *KRAS*, and *RB1* also showed higher mutation rates in the high‐risk group, the differences between the two groups did not reach statistical significance. To further characterize the association between *TP53* alteration and the imaging‐defined risk phenotype, we analyzed the distribution of *TP53* mutation subtypes (Table S5). While *TP53* mutations were more frequent in the high‐risk group, the relative proportions of specific mutation subtypes (e.g., missense, nonsense) did not differ significantly between groups.

## 3 Discussion

The advent of neoadjuvant therapy represents a paradigm shift in NSCLC immunotherapy [6, 26, 27], highlighting the critical need for non‐invasive imaging biomarkers to accurately identify pCR. Immunotherapy can induce atypical radiological patterns, such as immune cell infiltration or central necrosis, that do not coincide with measurable tumor shrinkage [28]. Consequently, size‐based criteria such as RECIST often fail to capture true pathological response [12, 29, 30], as illustrated in our representative case analysis (Figure 6B). To address these issues, we developed a deep learning model following a “foundation model pre‐training, downstream fine‐tuning” paradigm. This approach enables accurate prediction of pCR from baseline CT images, leveraging standardized imaging and robust feature representations to detect radiological signatures associated with treatment response that are not accessible through conventional assessments.

Previous studies have shown that deep learning models are effective tools to predict the efficacy of NCI in NSCLC (summarized in Table S4). For example, She et al. developed a model using CT images from 142 patients, which achieved an AUC of 0.73‐0.75 [31]. Ye et al. developed the LUNAI‐fCT model based on pre‐treatment CT images from 225 NSCLC patients, which obtained an AUC of 0.866 [32]. Zheng et al. developed the NeoPred model with dual‐phase CT images and clinical variables from 509 patients, achieving AUC values of 0.772‐0.787 [33]. However, these earlier models were trained from scratch on relatively limited datasets, which increases the risk of overfitting and may limit the extraction of robust, transferable features required for reliable multi‐center generalization. Notably, Ye et al. established the LC‐NICER system, which integrates radiomics, deep learning, and habitat imaging features from longitudinal CT data of 534 patients, yielding an AUC of 0.870 in the external test cohort [34]. However, despite incorporating a foundation model, the efficacy of the deep learning features was constrained by the absence of task‐specific fine‐tuning. In contrast, our study proposed a MTST model built upon a pretrained lung CT foundation model to predict pCR of NCI in NSCLC patients. Leveraging large‐scale pretraining and downstream fine‐tuning, the proposed model demonstrated strong performance, with AUCs ranging from 0.75 to 0.87 across the multi‐center external cohorts. These results highlight its potential utility for identifying patients most likely to benefit from NCI.

Domain specific adaptation is critical to apply foundation models to specialized clinical decision task [16]. Although generalist foundation models like VoCo are powerful, their pre‐training on diverse medical data may not fully capture the fine‐grained feature representations required for highly specific downstream tasks such as pCR prediction [17, 18, 19]. To address this limitation, we used a relatively large dataset (n = 6,643) to adapt the pre‐trained VoCo foundation model to the lung cancer domain using SSL strategy, thereby building a more robust and task‐relevant feature backbone. During the fine‐tuning phase, we further introduced an innovative multi‐task synergistic framework in which the segmentation branch provides spatial guidance to the classification branch. This design ensures that classification focuses on tumor‐relevant regions rather than background structures. The results suggest that co‐training the segmentation enhances pCR classification by improving spatial precision, while high‐level semantic features from the classification task provide additional supervisory signals that refine tumor boundary detection. Together, this two‐stage strategy‐combining deep domain adaptation with synergistic multi‐task fine‐tuning‐offers a robust pathway for transforming generalist foundation models into high‐performance, domain‐specific clinical prediction tools.

High‐quality imaging is a critical determinant of deep learning performance, and CT slice thickness is a major factor influencing volumetric spatial resolution [23, 35, 36]. To address the substantial slice‐thickness heterogeneity inherent in multi‐center datasets and to improve downstream predictive performance, we developed the ViMCT model to perform 3D chest CT super‐resolution. As shown in Figure 3, ViMCT achieved superior reconstruction quality compared with TIP and demonstrated a statistically significant advantage over the recent state‐of‐the‐art TVSR model [23]. Beyond its role in this study, ViMCT represents a generalizable tool that can mitigate the known performance degradation encountered when models rely on thick‐slice CT, thereby supporting broader AI applications in clinical imaging. Despite these strengths, inter‐center variations in CT acquisition and reconstruction protocols may persist after slice‐thickness standardization, potentially contributing to performance fluctuations across cohorts. Future studies should therefore further evaluate the robustness of synthetic thin‐slice images across diverse multicenter protocols, incorporating large‐scale external qualitative assessments to confirm their clinical reliability and interpretability in real‐world settings.

While deep learning models have shown powerful performance in medical image analysis, their clinical translation is often hindered their inherent “black box” nature [37, 38, 39]. To enhance model interpretability, the multitask design provides spatial guidance to the classification branch through tumor segmentation (Figure S5), indicating that predictive information is primarily concentrated in tumor‐related structural patterns. This observation is further supported by representative Grad‐CAM++ visualizations. Biologically, these attention patterns may be consistent with differences in the tumor microenvironment. Although these findings provide valuable biological insights, future research integrating explainability methods with radiologic‐pathologic correlation remains essential to further define the imaging features driving these predictions.

To explore the biological plausibility of the MTST model, we constructed a surrogate cohort of 110 NSCLC patients who underwent primary surgical resection. This cohort was designed to approximate the baseline biological status of patients receiving NCI. The results (Figure 7) suggest that the high‐risk imaging phenotype identified by the MTST model was not significantly associated with TMB. Although this analysis was not designed to re‐evaluate TMB as a treatment‐response biomarker, the absence of a significant association may suggest that the imaging‐derived risk phenotype captures biological information not fully reflected by overall mutational burden. Among common driver alterations, *TP53* was the only gene showing a statistically significant difference in mutation frequency between high‐risk and low‐risk group (P<0.05). In contrast, TMB did not differ between our high‐ and low‐risk groups, effectively decoupling *TP53* status from TMB in this setting. These findings demonstrate that the MTST model captures a radiographic phenotype strongly associated with *TP53* mutation status, likely reflecting morphological manifestations of the aggressive biological behavior driven by this alteration. This robust radiogenomic association enhances both the biological plausibility and the clinical interpretability of the model's predictions, supporting its potential for trustworthy decision‐making in NCI.

Our study not only introduces a high‐performance deep learning framework, but also derives a non‐invasive, foundation model‐based chest CT biomarker for risk stratification of pathological response. This biomarker offers three distinct advantages over current standards. First, it is entirely non‐invasive, relying solely on routine CT scans and thereby avoiding the risks and costs associated with tissue biopsies [40]. Second, Unlike tissue‐based assays such as PD‐L1, which are limited by spatial heterogeneity and sampling bias [41, 42], our CT biomarker interrogates the entire three‐dimensional tumor microenvironment, providing a holistic assessment of tumor biology. Third, most importantly for clinical translation, the CT biomarker enables prediction prior to therapeutic intervention. Notably, although the model was trained using pCR as the primary endpoint, its operating characteristics showed higher specificity than sensitivity across the validation cohorts. This indicates the model is particularly adept at identifying patients at high risk of having residual disease (non‐pCR). Therefore, its primary clinical value is not as a ‘rule‐in’ test for pCR, but as a non‐invasive baseline risk‐stratification tool to identify patients at higher risk of residual disease. This upfront risk stratification has the potential to identify likely non‐responders, preventing ineffective treatments and guiding more efficient, personalized therapeutic strategies. Importantly, this foundation‐model‐based CT biomarker is designed to complement, not replace, existing clinical and genomic markers, offering a more comprehensive and accurate assessment of pCR and enhancing the precision of NSCLC treatment planning [43, 44].

While this study provides promising results, several limitations must be addressed. First, the retrospective design and the use of external cohorts within a shared clinical trial framework (NCT04766515) may introduce residual selection bias. Although this design supports assessment of cross‐center reproducibility, it may not fully capture real‐world clinical diversity, and further validation in broader non‐trial populations is warranted. Second, we observed comparatively lower performance in Validation Cohort 1, which may be attributable to the high proportion of patients with clinical staged IIIB/IIIC resectable NSCLC in this cohort. This highlights the need for dedicated subgroup analyses in future work to assess the model's generalizability across different clinical stages and to optimize robustness. Third, as the training data were primarily derived from patient Chinese cohorts, model's performance across diverse ethnic populations and healthcare systems remains uncertain and requires further validation. Fourth, the observed association between the imaging‐defined high‐risk phenotype and *TP53* status does not establish a direct mechanistic link. Although this imaging phenotype may capture biological information complementary to existing biomarkers, these findings are hypothesis‐generating, and further multi‐omic studies are required to define the underlying mechanisms. Fifth, while our imaging‐based approach establishes an independent predictive baseline, multimodal integration was constrained by the lack of uniform genomic data in these retrospective cohorts. Future research should explore multimodal fusion strategies, integrating imaging features with the clinical and genomic variables, to enhance predictive accuracy and clinical utility.

## 4 Methods

### 4.1 Study Data and Patient Cohort

This multi‐center, retrospective observational study investigated the treatment response in resectable NSCLC patients receiving NCI across five hospitals. Inclusion criteria were as follows: (1) histologically confirmed NSCLC, clinically staged IB to IIIC; (2) administration of NCI followed by surgical resection during the study period; and (3) postoperative pathological evaluation of the tumor and lymph nodes as per IASLC guidelines. Exclusion criteria included: (1) absence of pre‐treatment CT images; (2) missing key clinical variables; or (3) presence of distant metastases or a history of other malignancies. Data completeness was assessed before analysis, and only patients with complete clinical and imaging data were included to ensure the validity and robustness of the study findings. Collected clinicopathological data included age, gender, smoking history, pre‐treatment clinical stage, pathological type, and post‐operative pathological response. The nineth edition of the IASLC TNM staging system was used for tumor staging [45].

This study was approved by the Ethics Committees of Fudan University Shanghai Cancer Center (No. 090977‐1); Shanghai Pulmonary Hospital, Tongji University (No. L20‐335‐2); Zhongshan Hospital Affiliated to Fudan University (No. B2021‐128); Tongji Hospital, Tongji Medical College of Huazhong University of Science and Technology (No. TJ‐IRB202412232); and Peking University Cancer Hospital (No. KC202509). The requirement for informed consent was waived due to the retrospective nature of the study and the anonymization of all patient data.

### 4.2 Pretreatment Evaluation and Treatment Protocol

Prior to NCI treatment, a comprehensive evaluation was conducted to diagnose and stage the tumor. This evaluation involved various preoperative examinations, including enhanced MRI of the head, contrast‐enhanced CT of the chest, abdominal ultrasound, whole‐body bone scintigraphy, or positron emission tomography‐CT. Additionally, pathological diagnosis was obtained through procedures such as tissue biopsy via bronchoscopy, ultrasound bronchoscopy‐guided transbronchial fine needle aspiration, or CT‐guided percutaneous puncture.

Patients typically received a standard NCI regimen consisting of platinum‐based chemotherapy combined with a PD‐1/PD‐L1 inhibitor (e.g., pembrolizumab or nivolumab). The chemotherapy regimen typically included cisplatin or carboplatin combined with paclitaxel or pemetred. Immunotherapy was administered every 3 weeks for up to four cycles. Surgical resection was performed 4–6 weeks after the completion of neoadjuvant therapy.

### 4.3 Pathological Evaluation

Postoperative pathological specimens were assessed by two senior pathologists. In accordance with IASLC guidelines, proficient pathologists assessed the postoperative pathological response of the primary tumor and lymph nodes in NSCLC patients who underwent NCI. The pCR was defined as the absence of viable tumor cells (ypT0 and ypN0) in both the tumor bed and lymph nodes.

### 4.4 ViMCT Model Development

In this study, we developed ViMCT, a model featuring a synergy between a 3D Swin Transformer and a Multiview Vision Mamba, to enhance the resolution of CT images. First, we used a 3D Swin‐Transformer Block as the encoder, continuing its proven capability in capturing 3D spatial dependencies and adeptly capturing complex global anatomical context and long‐range dependencies within the volumetric CT data [23]. Second, the Mask Token Add Module was used to dynamically supplement information in feature‐deficient regions. Finally, we innovatively used a Multiview Vision Mamba Block as the decoder [46]. This module leverages its gated state update mechanism to precisely extract local details, such as texture and edges of lung tissue, from multi‐planar reconstruction views. These modules operate via a synergistic mechanism: the 3D Swin Transformer provides 3D structural constraints for the Mamba, ensuring the anatomical plausibility of the extracted multi‐view local details. Concurrently, the fine‐grained features extracted by Mamba from multiple views are fed back to the 3D Swin Transformer to correct potential local feature deficiencies.

For performance comparison, we generated synthetic thin‐slice CT using Trilinear Interpolation (via SimpleITK v2.0, https://simpleitk.org/doxygen/v2_0/html/) to serve as a comparative baseline. The TVSR model was obtained from its GitHub repository (https://github.com/smilenaxx/CTHNet‐for‐CT‐Slice‐Thickness‐Reduction) for thin‐slice CT synthesis. Finally, we compared the performance of ViMCT against TIP and TVSR on two independent validation sets.

### 4.5 Image Acquisition and Tumor Segmentation

We acquired baseline chest CT images for neoadjuvant treatment from the five centers. The acquisition parameters for these CT scans, particularly slice thickness, were heterogeneous across the centers, ranging from 1 to 5 mm. To standardize these heterogeneous data, we employed the ViMCT model to process all original thick‐slice CTs from the study cohort into high‐quality synthetic thin‐slice CTs. Three radiologists used ITK‐SNAP software (http://www.itksnap.org) to delineate the region of interest (ROI) layer‐by‐layer on these standardized axial CT images, with verification and adjustment in the sagittal and coronal reconstructions. For each case, the primary lesion was selected as the target lesion. The ROI was meticulously delineated along the tumor's perimeter to ensure comprehensive inclusion of the entire tumor while carefully excluding non‐tumor tissues, such as blood vessels, trachea, and ribs. Any disagreements among the radiologists were resolved through discussion to achieve consensus.

### 4.6 MTST Model Development

VoCo is an SSL framework designed for 3D medical imaging [22]. Inspired by this, this study used pretrained VoCo with 160K CT volumes as the starting point to build our Lung CT Foundation Model. Specifically, we utilized VoCo's framework to perform domain‐adaptive fine‐tuning on 6,643 chest CT images from Fudan University Shanghai Cancer Center (with inclusion not limited by age, gender, or presence of pathology). This aimed to enable the model to specifically learn pulmonary anatomy and imaging features while retaining VoCo's robust 3D spatial context modeling capabilities. The ultimate value of this Lung CT Foundation Model was realized in its adaptation to downstream clinical tasks. We transferred and fine‐tuned this model into an MTST framework for precise NCI treatment response risk stratification. The MTST framework simultaneously performs two related tasks: an upper branch executes the segmentation task (Segmentation Output) via multiple 3D ST Blocks, Patch Merging, and Res‐Blocks to precisely delineate the tumor region; a lower branch executes the classification task to predict treatment response risk. A key design feature is the interaction between the tasks: the voxel‐wise map generated by the segmentation branch is integrated into the classification branch, enabling the model to focus on features within the tumor region for risk prediction. Finally, the classification branch fuses feature via Avg Pooling and Fully Connected Layers to output the final pCR prediction (framework flowchart shown in Figure S1). The multi‐task learning loss function was formulated as:

$$L_{MTST}=\lambda L_{seg}+\left(1-\lambda \right)L_{clf}$$

where *L*_seg_ denotes the segmentation loss and *L*_clf_ represents the classification loss. Specifically, Dice loss was used for segmentation $L_{\mathrm{seg}}=1-\mathrm{Dice}\left(y_{\mathrm{seg}},{\hat{y}}_{\mathrm{seg}}\right),$ and binary cross‐entropy was used for pCR classification $L_{clf}=-\log\left({\hat{y}}_{clf}\right)-\left(1-y_{clf}\right)\log\left(1-{\hat{y}}_{clf}\right)$. The weighting coefficient λ∈[0,1] balances the contribution between segmentation and classification branches. In this study, λ was empirically set to 0.5 to equally emphasize both tasks during optimization. To evaluate the performance of the MTST framework, we compared it with single task models of segmentation and classification.

The model was developed using full volumetric pretreatment chest CT data represented in Hounsfield units. Before model training, voxel intensities were clipped to a fixed range of [−1000, 600] HU and linearly normalized to [0, 1] according to the following formula:

$$I_{\mathrm{norm}}=\frac{I_{\mathrm{orig}}+1000}{1600}$$

where I_orig_ denotes original CT image and I_norm_ denotes normalized image. The study cohort was collected from five centers to reflect real‐world clinical practice, and acquisition protocols, including the contrast phase, were heterogeneous. The dataset consisted predominantly of arterial‐phase scans, though this was not a strict selection criterion.

### 4.7 Model Evaluation and Statistical Analysis

For the ViMCT model, we evaluated performance using PSNR, SSIM, and MSE. For the segmentation performance of the MTST model, we used the Dice coefficient, Jaccard coefficient, and HD95. For the classification performance of the MTST model, we first calculated the ROC curve and the AUC to evaluate the model's ability to discriminate between pCR and non‐pCR groups. Second, we quantified overall predictive reliability using AUC, accuracy, sensitivity, specificity, PPV, and NPV. Third, DCA was employed to validate the model's clinical utility by assessing the Net Benefit across different decision thresholds. Finally, calibration curves were used to assess the consistency between the model's predicted probabilities and observed event frequencies.

For statistical analysis, categorical variables in the baseline data were compared using the Chi‐squared (X^2^) test or Fisher's exact test. Continuous variables were compared using the Student's t‐test or Mann‐Whitney U test, as appropriate for their distribution. Continuous data were presented as mean ± standard deviation (SD) or median (interquartile range), while categorical data were presented as counts (percentages). The DeLong test was used to assess differences between AUCs.

### 4.8 Radiogenomic Association Analysis

For the biological basis investigation, we retrospectively enrolled NSCLC patients who underwent direct surgical resection at our center from March 2020 to March 2024. Inclusion criteria were as follows: (1) pathological diagnosis of NSCLC; (2) underwent direct surgical resection during the study period without any prior therapeutic intervention; (3) complete pre‐operative CT images were available; and (4) sufficient specimen materials (paraffin‐embedded blocks and peripheral blood) were available for genetic testing. The detection method was Illumina NextSeq high‐throughput sequencing, using the Human 520‐gene mutation detection kit from Burning Rock Biotech (Guangzhou, China). Specific detection methods and the full list of genes are detailed in the Supplementary Materials. We retrospectively collected data on mutated genes, gene mutation types, and TMB values.

The criteria for including genes for discussion were: being a recognized driver gene in NSCLC, or having a mutation rate >3% in either the high‐ or low‐risk group. An empirical threshold of >3% was used to balance the inclusion of biologically relevant genes with the need for statistical stability in our modest‐sized radiogenomic cohort. For statistical analysis, categorical variables were compared using the Fisher's exact test. Continuous variables were compared using the unpaired two‐tailed Wilcoxon rank‐sum test, with P‐values used to indicate significant differences in the figures.

## Author Contributions

**Xiao Bao**: investigation, validation, resources. **Xiaodan Ye**: validation, supervision, resources. **Qin Peng**: methodology, data curation, investigation, writing – review and editing. **Tong Tong**: supervision, resources. **Ahmet Görkem Er**: methodology, writing – review and editing. **Yajia Gu**: conceptualization, resources, project administration, writing – review and editing. **Jing Gong**: conceptualization, methodology, formal analysis, funding acquisition, project administration, resources, writing – review and editing. **Shuchang Zhou**: methodology, investigation, validation, funding acquisition, resources. **Mirabela Rusu**: writing – review and editing. **Mailin Chen**: methodology, resources, project administration, funding acquisition, validation, writing – review and editing. **Yanglan Xu**: methodology, data curation, investigation, writing – original draft, visualization, conceptualization.

## Conflicts of Interest

The authors declare no conflicts of interest.

## Supporting information




**Supporting File**: advs75933‐sup‐0001‐SuppMat.docx.

## Data Availability

The datasets generated and/or analyzed during the current study are not publicly available due to ethical restrictions related to the protection of participants’ privacy but are available from the corresponding author on reasonable request.

## References

[1] H. Sung , J. Ferlay , R. L. Siegel , et al., “Global Cancer Statistics 2020: GLOBOCAN Estimates of Incidence and Mortality Worldwide for 36 Cancers in 185 Countries,” CA: A Cancer Journal for Clinicians 71, no. 3 (2021): 209–249. 33538338 10.3322/caac.21660

[2] R. L. Siegel , K. D. Miller , H. E. Fuchs , and A. Jemal , “Cancer Statistics, 2022,” CA: A Cancer Journal for Clinicians 72, no. 1 (2022): 7–33. 35020204 10.3322/caac.21708

[3] A. Lahiri , A. Maji , P. D. Potdar , et al., “Lung Cancer Immunotherapy: Progress, Pitfalls, and Promises,” Molecular Cancer 22, no. 1 (2023): 40, 10.1186/s12943-023-01740-y. 36810079 PMC9942077

[4] L. Gandhi , D. Rodríguez‐Abreu , S. Gadgeel , et al., “Pembrolizumab Plus Chemotherapy in Metastatic Non–Small‐Cell Lung Cancer,” New England Journal of Medicine 378, no. 22 (2018): 2078–2092, 10.1056/NEJMoa1801005. 29658856

[5] S. J. Antonia , A. Villegas , D. Daniel , et al., “Overall Survival With Durvalumab After Chemoradiotherapy in Stage III NSCLC,” New England Journal of Medicine 379, no. 24 (2018): 2342–2350, 10.1056/NEJMoa1809697. 30280658

[6] P. M. Forde , J. Spicer , S. Lu , et al., “Neoadjuvant Nivolumab Plus Chemotherapy in Resectable Lung Cancer,” New England Journal of Medicine 386, no. 21 (2022): 1973–1985, 10.1056/NEJMoa2202170. 35403841 PMC9844511

[7] M. Reck , J. Remon , and M. D. Hellmann , “First‐Line Immunotherapy for Non–Small‐Cell Lung Cancer,” Journal of Clinical Oncology 40, no. 6 (2022): 586–597, 10.1200/JCO.21.01497. 34985920

[8] S. Rosner , C. Liu , P. M. Forde , and C. Hu , “Association of Pathologic Complete Response and Long‐Term Survival Outcomes Among Patients Treated With Neoadjuvant Chemotherapy or Chemoradiotherapy for NSCLC: A Meta‐Analysis,” JTO Clinical and Research Reports 3, no. 9 (2022): 100384. 36118131 10.1016/j.jtocrr.2022.100384 PMC9472066

[9] B. Hui , X. Wang , X. Wang , et al., “Multicenter Retrospective Cohort Study,” International Journal of Surgery 109, no. 8 (2023): 2286–2292, 10.1097/JS9.0000000000000455. 37161431 PMC10442100

[10] H. Deng , Y. Zhao , X. Cai , et al., “PD‐L1 Expression and Tumor Mutation Burden as Pathological Response Biomarkers of Neoadjuvant Immunotherapy for Early‐Stage Non‐Small Cell Lung Cancer: A Systematic Review and Meta‐Analysis,” Critical Reviews in Oncology/Hematology 170 (2022): 103582, 10.1016/j.critrevonc.2022.103582. 35031441

[11] M. L. Maitland , J. Wilkerson , S. Karovic , et al., “Enhanced Detection of Treatment Effects on Metastatic Colorectal Cancer With Volumetric CT Measurements for Tumor Burden Growth Rate Evaluation,” Clinical Cancer Research 26, no. 24 (2020): 6464–6474, 10.1158/1078-0432.CCR-20-1493. 32988968 PMC8170504

[12] M. Provencio , E. Nadal , A. Insa , et al., “Neoadjuvant Chemotherapy and Nivolumab in Resectable Non‐Small‐Cell Lung Cancer (NADIM): an Open‐Label, Multicentre, Single‐Arm, Phase 2 Trial,” The Lancet Oncology 21, no. 11 (2020): 1413–1422, 10.1016/S1470-2045(20)30453-8. 32979984

[13] M. Chen , S. J. Copley , P. Viola , H. Lu , and E. O. Aboagye , “Radiomics and Artificial Intelligence for Precision Medicine in Lung Cancer Treatment,” Seminars in Cancer Biology 93 (2023): 97–113, 10.1016/j.semcancer.2023.05.004. 37211292

[14] P. Rajpurkar , E. Chen , O. Banerjee , and E. J. Topol , “AI in Health and Medicine,” Nature Medicine 28, no. 1 (2022): 31–38, 10.1038/s41591-021-01614-0. 35058619

[15] X. Wang , Y. Jiang , H. Chen , et al., “Cancer Immunotherapy Response Prediction From Multi‐Modal Clinical and Image Data Using Semi‐Supervised Deep Learning,” Radiotherapy and Oncology 186 (2023): 109793, 10.1016/j.radonc.2023.109793. 37414254

[16] M. Moor , O. Banerjee , Z. S. H. Abad , et al., “Foundation Models for Generalist Medical Artificial Intelligence,” Nature 616, no. 7956 (2023): 259–265, 10.1038/s41586-023-05881-4. 37045921

[17] Y. Bian , J. Li , C. Ye , X. Jia , and Q. Yang , “Artificial Intelligence in Medical Imaging: From Task‐Specific Models to Large‐Scale Foundation Models,” Chinese Medical Journal 138, no. 6 (2025): 651–663, 10.1097/CM9.0000000000003489. 40008785 PMC11925424

[18] Z. Huang , H. Wang , Z. Deng , et al., “STU‐Net: Scalable and Transferable Medical Image Segmentation Models Empowered by Large‐scale Supervised Pre‐training,” arXiv (2023), 10.48550/arXiv.2304.06716.

[19] J. Ma , Y. He , F. Li , L. Han , C. You , and B. Wang , “Segment Anything in Medical Images,” Nature Communications 15, no. 1 (2024): 654, 10.1038/s41467-024-44824-z. PMC10803759 38253604

[20] J. Xiang , X. Wang , X. Zhang , et al., “A Vision–Language Foundation Model for Precision Oncology,” Nature 638, no. 8051 (2025): 769–778, 10.1038/s41586-024-08378-w. 39779851 PMC12295649

[21] F. Haghighi , M. B. Gotway , and J. Liang , “Large‐Scale Benchmarking and Boosting Transfer Learning for Medical Image Analysis,” Medical Image Analysis 102 (2025): 103487. 40117988 10.1016/j.media.2025.103487

[22] L. Wu , J. Zhuang , and H. Chen , “Single‐Step Latent Diffusion for Underwater Image Restoration,” IEEE Transactions on Pattern Analysis and Machine Intelligence (2025): 1–11, 10.1109/TPAMI.2025.3599775. 40815580

[23] P. Yu , H. Zhang , D. Wang , et al., “Spatial Resolution Enhancement Using Deep Learning Improves Chest Disease Diagnosis Based on Thick Slice CT,” npj Digital Medicine 7 (2024): 335, 10.1038/s41746-024-01338-8. 39580609 PMC11585608

[24] A. Hatamizadeh , V. Nath , Y. Tang , D. Yang , H. R. Roth , and D. Xu , “Swin UNETR: Swin Transformers for Semantic Segmentation of Brain Tumors in MRI Images,” arXiv (2022), 10.48550/arXiv.2201.01266.

[25] E. A. Eisenhauer , P. Therasse , J. Bogaerts , et al., “New Response Evaluation Criteria in Solid Tumours: Revised RECIST Guideline (version 1.1),” European Journal of Cancer 45, no. 2 (2009): 228–247, 10.1016/j.ejca.2008.10.026. 19097774

[26] H. Wakelee , M. Liberman , T. Kato , et al., “Perioperative Pembrolizumab for Early‐Stage Non–Small‐Cell Lung Cancer,” New England Journal of Medicine 389, no. 6 (2023): 491–503, 10.1056/NEJMoa2302983. 37272513 PMC11074923

[27] J. V. Heymach , D. Harpole , T. Mitsudomi , et al., “Perioperative Durvalumab for Resectable Non–Small‐Cell Lung Cancer,” New England Journal of Medicine 389, no. 18 (2023): 1672–1684, 10.1056/NEJMoa2304875. 37870974

[28] V. L. Chiou and M. Burotto , “Pseudoprogression and Immune‐Related Response in Solid Tumors,” Journal of Clinical Oncology 33, no. 31 (2015): 3541–3543, 10.1200/JCO.2015.61.6870. 26261262 PMC4622096

[29] L. Seymour , J. Bogaerts , A. Perrone , et al., “iRECIST: Guidelines for Response Criteria for use in Trials Testing Immunotherapeutics,” The Lancet Oncology 18, no. 3 (2017): e143–e152, 10.1016/S1470-2045(17)30074-8. 28271869 PMC5648544

[30] J. Kang , C. Zhang , and W. Z. Zhong , “Neoadjuvant Immunotherapy for Non–Small Cell Lung Cancer: State of the Art,” Cancer Communications 41, no. 4 (2021): 287–302, 10.1002/cac2.12153. 33689225 PMC8045926

[31] Y. She , B. He , F. Wang , et al., “Multicentre Study,” eBioMedicine 86 (2022): 104364, 10.1016/j.ebiom.2022.104364. 36395737 PMC9672965

[32] G. Ye , G. Wu , Y. Qi , et al., “Non‐Invasive Multimodal CT Deep Learning Biomarker to Predict Pathological Complete Response of Non‐Small Cell Lung Cancer Following Neoadjuvant Immunochemotherapy: A Multicenter Study,” Journal for ImmunoTherapy of Cancer 12, no. 9 (2024): 009348, 10.1136/jitc-2024-009348. PMC11409329 39231545

[33] J. Zheng , Z. Yan , R. Wang , et al., “NeoPred: Dual‐Phase CT AI Forecasts Pathologic Response to Neoadjuvant Chemo‐Immunotherapy in NSCLC,” Journal for ImmunoTherapy of Cancer 13, no. 5 (2025): 011773, 10.1136/jitc-2025-011773. PMC12163334 40449955

[34] G. Ye , Z. Wei , C. Han , et al., “AI‐Derived Longitudinal and Multi‐Dimensional CT Classifier for Non‐Small Cell Lung Cancer to Optimize Neoadjuvant Chemoimmunotherapy Decision: A Multicentre Retrospective Study,” eClinicalMedicine 89 (2025): 103551, 10.1016/j.eclinm.2025.103551. 41127561 PMC12538916

[35] C. Peng , W. A. Lin , H. Liao , R. Chellappa , and S. K. Zhou , “SAINT: Spatially Aware Interpolation NeTwork for Medical Slice Synthesis,” arXiv (2020), 10.48550/arXiv.2001.00704.

[36] R. Berenguer , M. D. R. Pastor‐Juan , J. Canales‐Vázquez , et al., “Radiomics of CT Features may be Nonreproducible and Redundant: Influence of CT Acquisition Parameters,” Radiology 288, no. 2 (2018): 407–415, 10.1148/radiol.2018172361. 29688159

[37] C. Rudin , “Stop Explaining Black Box Machine Learning Models for High Stakes Decisions and use Interpretable Models Instead,” Nature Machine Intelligence 1, no. 5 (2019): 206–215, 10.1038/s42256-019-0048-x. PMC9122117 35603010

[38] E. J. Topol , “High‐Performance Medicine: The Convergence of Human and Artificial Intelligence,” Nature Medicine 25, no. 1 (2019): 44–56, 10.1038/s41591-018-0300-7. 30617339

[39] E. H. Houssein , A. M. Gamal , E. M. G. Younis , and E. Mohamed , “Explainable Artificial Intelligence for Medical Imaging Systems Using Deep Learning: A Comprehensive Review,” Cluster Computing 28, no. 7 (2025): 469, 10.1007/s10586-025-05281-5.

[40] W. J. Heerink , G. H. de Bock , G. J. de Jonge , H. J. M. Groen , R. Vliegenthart , and M. Oudkerk , “Complication Rates of CT‐Guided Transthoracic Lung Biopsy: Meta‐Analysis,” European Radiology 27, no. 1 (2017): 138–148, 10.1007/s00330-016-4357-8. 27108299 PMC5127875

[41] K. I. Zhou , B. Peterson , A. Serritella , et al., “Spatial and Temporal Heterogeneity of PD‐L1 Expression and Tumor Mutational Burden in Gastroesophageal Adenocarcinoma at Baseline Diagnosis and After Chemotherapy,” Clinical Cancer Research 26, no. 24 (2020): 6453–6463, 10.1158/1078-0432.CCR-20-2085. 32820017 PMC7744325

[42] A. Haragan , J. K. Field , M. P. A. Davies , C. Escriu , A. Gruver , and J. R. Gosney , “Heterogeneity of PD‐L1 Expression in Non‐Small Cell Lung Cancer: Implications for Specimen Sampling in Predicting Treatment Response,” Lung Cancer 134 (2019): 79–84, 10.1016/j.lungcan.2019.06.005. 31320000 PMC6658831

[43] W. He , W. Huang , L. Zhang , X. Wu , S. Zhang , and B. Zhang , “Radiogenomics: Bridging the Gap Between Imaging and Genomics for Precision Oncology,” MedComm 5, no. 9 (2020): 722. 10.1002/mco2.722 PMC11381657 39252824

[44] R. S. Vanguri , J. Luo , A. T. Aukerman , et al., “Multimodal Integration of Radiology, Pathology and Genomics for Prediction of Response to PD‐(L)1 Blockade in Patients With Non‐Small Cell Lung Cancer,” Nature Cancer 3, no. 10 (2022): 1151–1164, 10.1038/s43018-022-00416-8. 36038778 PMC9586871

[45] H. Asamura , K. K. Nishimura , D. J. Giroux , et al., “IASLC Lung Cancer Staging Project: The New Database to Inform Revisions in the Ninth Edition of the TNM Classification of Lung Cancer,” Journal of Thoracic Oncology 18, no. 5 (2023): 564–575, 10.1016/j.jtho.2023.01.088. 36773775

[46] A. Gu and T. Dao , “Mamba: Linear‐Time Sequence Modeling with Selective State Spaces,” arXiv (2023), 10.48550/arXiv.2312.00752.

