# Supplementary material for: A Foundation Model Based CT Biomarker for Non‐Invasive Prediction of Response to Neoadjuvant Immunochemotherapy in Non‐Small Cell Lung Cancer
Source: Adv Sci (Weinh). 2026 Jun 2:e75933. Online ahead of print. doi: 10.1002/advs.75933 (PMC13337109; doi:10.1002/advs.75933)
Supplement: Supplementary file 1 — Supporting File: advs75933‐sup‐0001‐SuppMat.docx. [file ADVS-9999-e75933-s001.docx]

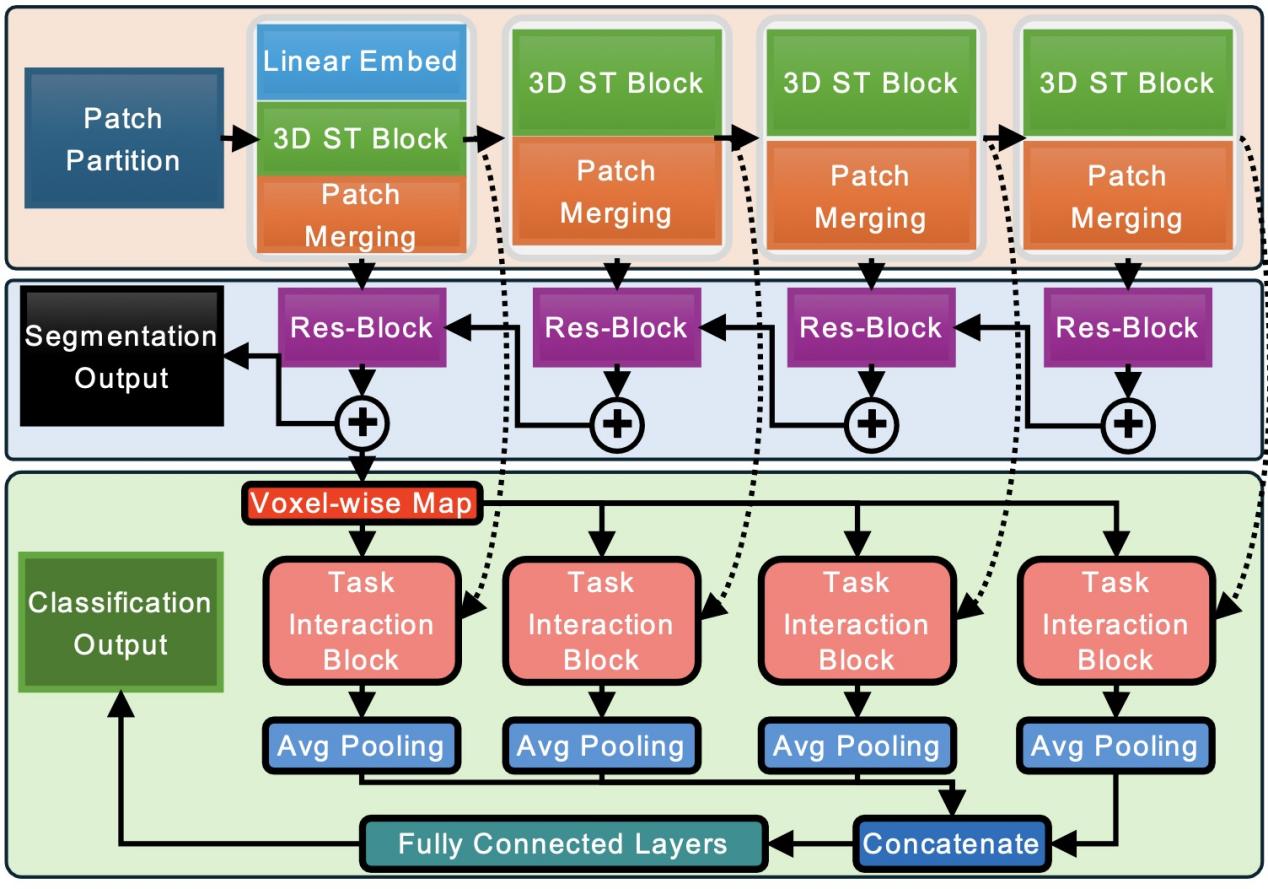


**Supplementary Figure 1. Architecture of the Multi-Task Swin-Transformer (MTST).**

The workflow initiates with Patch Partition and Linear Embed, followed by a shared encoder comprising 3D ST Blocks and Patch Merging. The segmentation branch utilizes Residual Blocks (Res-Block) to generate the Segmentation Output. Crucially, the resulting Voxel-wise Map is integrated into the classification branch via Task Interaction Blocks for spatial guidance. Finally, multi-scale features are processed via Average Pooling (Avg Pooling), fused using Concatenate, and passed through Fully Connected Layers to yield the final Classification Output (pCR risk score).

Abbreviations: ST, Swin-Transformer; pCR, pathological complete response.

**
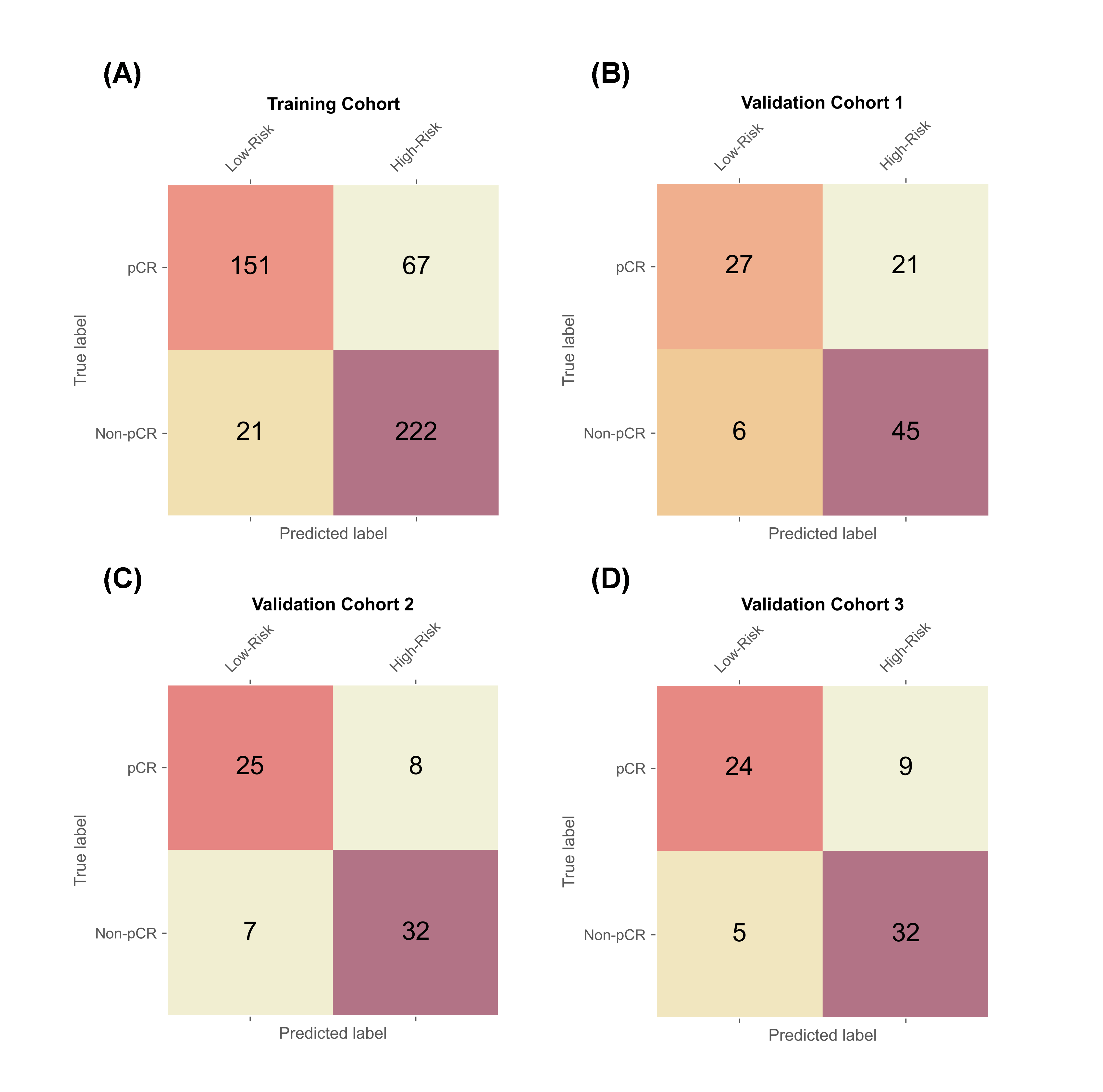
**

**Supplementary Figure 2. Confusion matrices of the MTST model.**

Panels (A) to (D) display the confusion matrices, comparing the ground truth clinical outcomes with the MTST model predictions across the training and three independent validation cohorts. The model stratifies patients into “Low-Risk” (predicted pCR) and “High-Risk” (predicted non-pCR).

Abbreviations: pCR, pathological complete response.

**
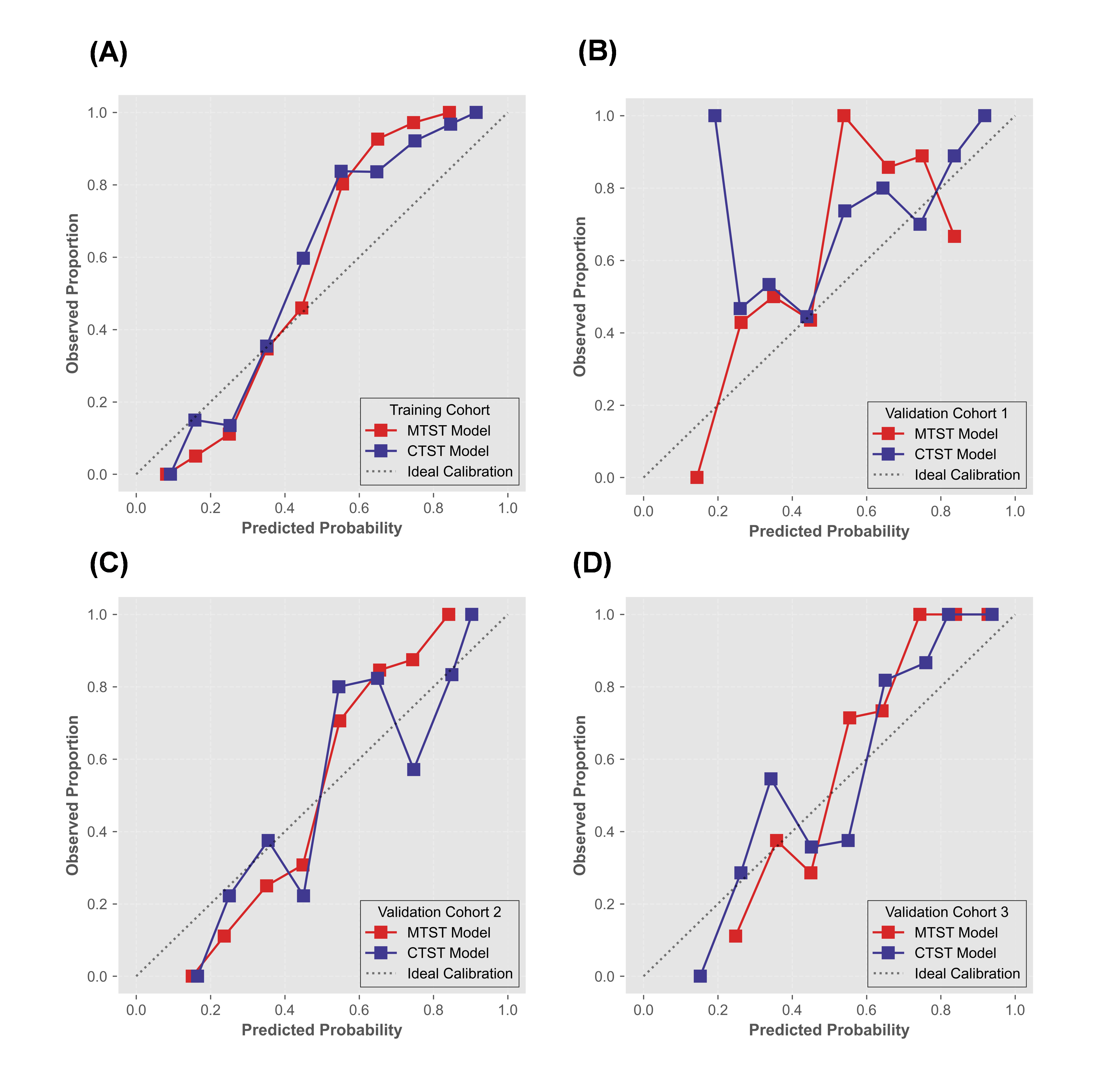
**

**Supplementary Figure 3. Comparison of model calibration performance.**

Panels (A) to (D) display the calibration curve analysis, comparing the calibration performance of the MTST model (red) and CTST model (blue) across the training and three independent validation cohorts. The gray dotted line represents ideal calibration. MTST consistently demonstrated superior calibration accuracy in all cohorts, showing closer alignment with the ideal line compared to the CTST model.

Abbreviations: MTST, Multi-Task Swin-Transformer; CTST, single Classification-Task Swin-Transformer.


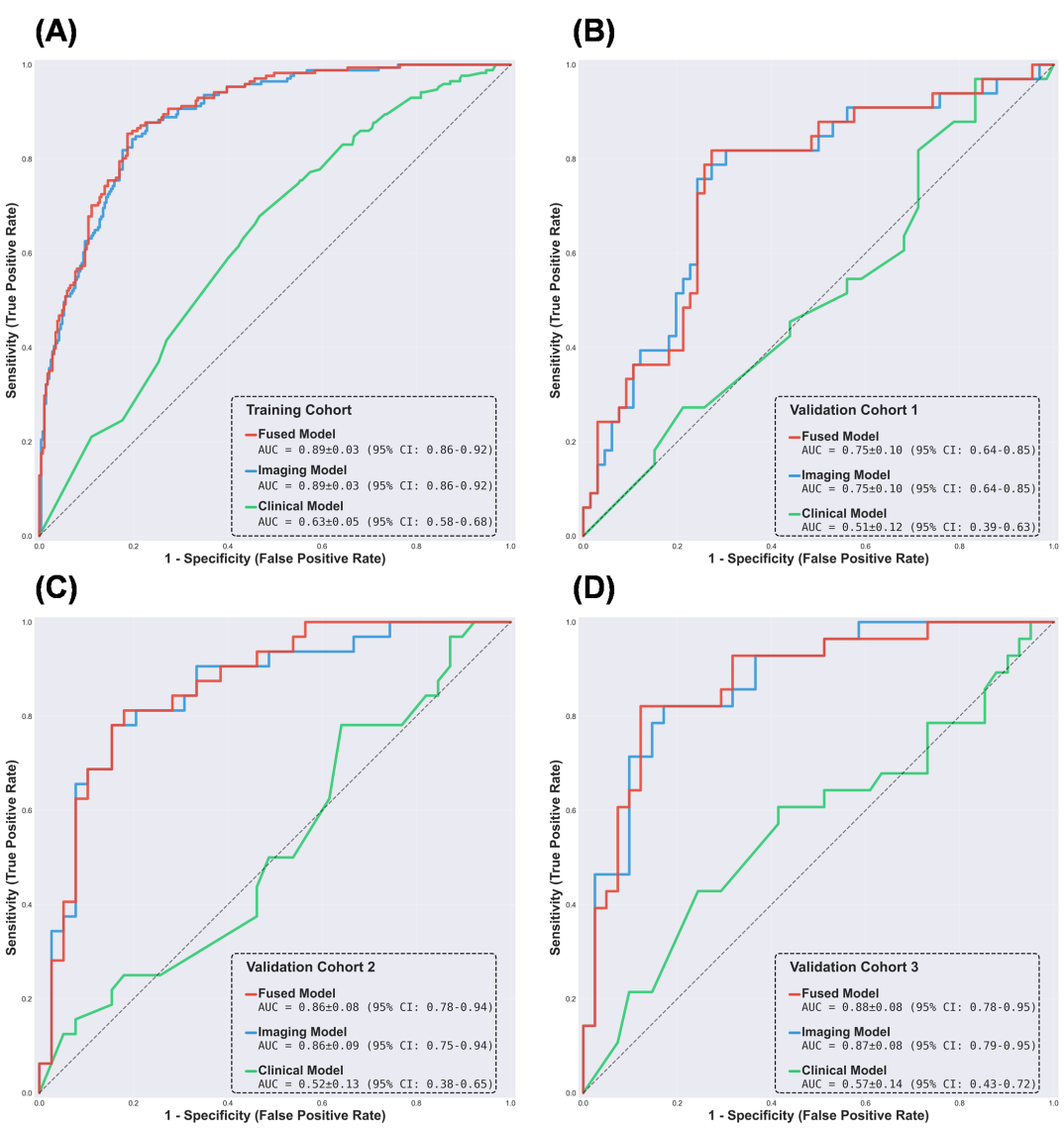


**Supplementary Figure 4. ROC curves for the clinical, imaging, and fusion models.**

1. Training cohort; (B) to (D) three independent multicenter validation cohorts. ROC curves compare the discriminative performance of clinical (green), imaging (blue), and fused (red) models. The substantial overlap between fused and imaging curves indicates that predictive power is primarily driven by the imaging score.

Abbreviations: AUC, area under the ROC curve.

**
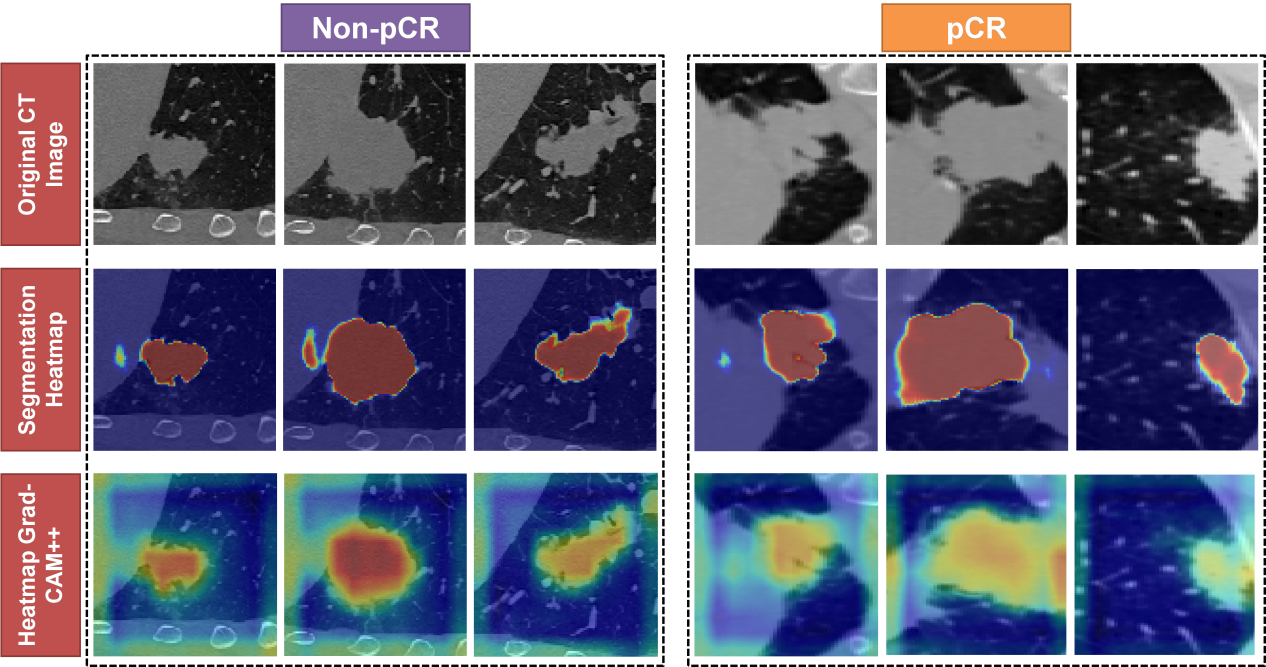
**

**Supplementary Figure 5. Visualization of Model Interpretability Using Grad-CAM++.** Representative non-pCR (left) and pCR (right) cases are shown. The segmentation probability maps (middle row) illustrate tumor localization by the model, while the Grad-CAM++ heatmaps (bottom row) show representative spatial attention patterns, with warm colors indicating regions of higher model attention.

**Supplementary Table 1. CT scanning parameters among multiple centers.**

| **Parameters** | **Center 1** | **Center 2** | **Center 3** | **Center 4** | **Center 5** |
| --- | --- | --- | --- | --- | --- |
| Manufacturer | GE/SIEMENS | GE/PHILIPS | GE/SIEMENS | SIEMENS/GE/UIH (United Imaging Healthcare) | GE/TOSHIBA |
| Tube Voltage, kVp | 120 | 110-130 | 110-130 | 120 | 100-120 |
| Tube Current | Automatic tube current modulation | Automatic tube current modulation | Automatic tube current modulation | Automatic tube current modulation | Automatic tube current modulation |
| Pitch | 0.60-1.20 | 0.60-1.20 | 0.60-1.40 | 1.00-1.40 | 1.00-1.50 |
| Collimation | 0.50-0.625 | 0.60-0.625 | 0.60 | 0.50-0.75 | 0.60-0.80 |
| Matrix | 512 × 512 | 512×512 | 512 × 512 | 512 × 512 | 512 × 512 |
| Pixel spacing, mm | 0.58-1.00 | 0.68-1.00 | 0.73-1.00 | 0.60-1.00 | 0.25-1.00 |
| Acquired slice thickness, mm | 0.60-2.00 | 1.00-5.00 | 0.60-5.00 | 3.00-5.00 | 1.00-5.00 |
| Reconstruction Kernel | LUNG/B31f | Lung/Y-sharp/iDose⁴ 3 | LUNG/B31f | LUNG/Standard | LUNG/Standard |

Note: Reconstruction kernel names are vendor-specific and are reported as provided by each center.

**Supplementary Table 2. Performance evaluation of MTST model in different cohorts.**

| Dataset | ACC | SEN | SPE | PPV | NPV |
| --- | --- | --- | --- | --- | --- |
| Training Cohort | 0.809  （95%CI 0.773-0.845） | 0.693 | 0.914 | 0.878 | 0.768 |
| Validation Cohort 1 | 0.727  (95%CI 0.639-0.815) | 0.5625 | 0.882 | 0.818 | 0.682 |
| Validation Cohort 2 | 0.792  (95%CI 0.698-0.886) | 0.758 | 0.821 | 0.781 | 0.800 |
| Validation Cohort 3 | 0.800  (95%CI 0.706-0.894) | 0.727 | 0.865 | 0.828 | 0.780 |

**Supplementary Table 3. Performance comparisons among different models in Cohort 1.**

| Method | AUC | 95% CI |
| --- | --- | --- |
| Traditional radiomics model ^[1]^ | 0.61±0.11 | 0.39-0.82 |
| Habitat imaging model ^[2]^ | 0.67±0.08 | 0.50-0.81 |
| Single-task model (CTST) | 0.68±0.06 | 0.58-0.77 |
| Multi-Task model (MTST) | 0.75±0.05 | 0.66-0.84 |

**Supplementary Table 4. Contextual comparison with representative prior CT-based studies.**

| Study | Method | Imaging Input | Dataset Size | AUC |
| --- | --- | --- | --- | --- |
| She et al. (2022) ^[3]^ | deep learning model | Baseline CT | 274 | 0.730-0.750 |
| Ye et al. (2024) ^[4]^ | deep learning model | Non-contrast baseline CT + Contrast-enhanced baseline CT | 225 | 0.866 |
| Zheng et al. (2025) ^[5]^ | deep learning model | Baseline CT + Postoperative CT | 509 | 0.772-0.787 |
| Ye et al. (2025) ^[6]^ | Radiomics + Habitat + Deep learning fusion model | Baseline CT + Postoperative CT | 534 | 0.870 |
| Our Method | Foundation model-based multitask network | Baseline CT | 702 | 0.750-0.870 |

**Supplementary Table 5. Comparison of *TP53* mutation status and subtype distribution between MTST-defined high- and low-risk groups**.

| ***TP53* Mutation** | **High-risk group** | **Low-risk group** | **P Value** |
| --- | --- | --- | --- |
| Mutation Status |  |  | 0.044* |
| Wild-type | 36 (48.6%) | 25 (69.4%) |  |
| Mutant | 38 (51.4%) | 11 (30.6%) |  |
| Mutation Type |  |  | 0.423 |
| Missense mutation | 24 (32.4%) | 6 (16.7%) |  |
| Nonsense mutation | 7 (9.5%) | 2 (5.6%) |  |
| Splice site mutation | 3 (4.1%) | 3 (8.3%) |  |
| Frameshift mutation | 3 (4.1%) | 0 (0.0%) |  |
| In-frame deletion | 1 (1.4%) | 0 (0.0%) |  |

Note: Data are presented as n (%). P values were calculated using Fisher’s exact test for overall *TP53* mutation status and for the distribution of mutation subtypes between the high- and low-risk groups.

**Supplementary Methods: Establishment of Clinical-Pathological Model and Fusion Model**

To establish a robust baseline for multi-center data, we developed a LASSO-penalized logistic regression model. Eight clinical features were discretized to mitigate noise (e.g., age dichotomized at 60 years). Although complex non-linear algorithms yielded higher training accuracy, we selected the linear approach to prevent severe overfitting and ensure external generalization. The final model achieved an AUC of 0.63 in the training cohort (n=461) and averaged 0.54 across three independent validation sets, with smoking history, gender, T/N stages, and pathological type identified as the primary predictors. Subsequently, to integrate the clinical baseline with the imaging prediction score, we developed a multimodal fusion model using Bayesian Model Averaging (BMA). We evaluated BMA against predefined linear weighting and data-driven logistic regression. While all fusion strategies significantly outperformed the standalone clinical model, BMA was selected for maximizing external generalization and minimizing overfitting. The final BMA model yielded an average AUC of 0.8311 across three independent validation sets. Weight optimization established a contribution ratio of 80.4% for imaging features versus 19.6% for clinical features. Notably, the multimodal fusion yielded only marginal performance gains over the standalone MTST approach, suggesting that the imaging score was the primary contributor to predictive performance in the fusion model.

**Supplementary Methods: 520-Gene Targeted Sequencing**

**Library Preparation and Sequencing.** DNA profiling was performed using the OncoScreen Plus 520-gene mutation detection kit (Burning Rock Biotech, Guangzhou, China). Targeted sequencing was executed on paired tumor tissue and control DNA using hybridization capture-based next-generation sequencing (NGS).

**Gene Panel Specifications.** The gene coverage profile was identical across all samples, consisting of: (1) full exonic coverage for 315 genes (including partial intronic coverage for 15 genes); (2) partial exonic coverage for 193 genes; (3) partial intronic coverage for 9 genes; and (4) specific polymorphic loci in 3 drug metabolism-related genes. The complete list of the 520 genes is provided in **Supplementary Data 1**.

**Quality Control and Limitations.** A minimum tumor cell content (tumor purity) of >20% was required for all tissue samples. The limit of detection for variants was established at 5% at a sequencing depth of 1000×. It is important to note that rare large-scale deletions or insertions may not be fully captured by this assay. Furthermore, the clinical interpretation of detected variants is subject to periodic re-evaluation based on evolving scientific evidence and database updates.

**References**

[1] Bao X, Peng Q, Bian D, Ni J, Zhou S, Zhang P, Gu Y, Gong J, Shi J. Short-term intra-and peri-tumoral spatiotemporal CT radiomics for predicting major pathological response to neoadjuvant chemoimmunotherapy in non-small cell lung cancer. European Radiology. 2025 Oct;35(10):6052-64.

[2] Peng Q, Xu Y, Shen L, Bao X, Zhou S, Ye X, et al. Habitat-based CT radiomics profiling spatial-temporal heterogeneity in resectable NSCLC predict pathological response to neoadjuvant chemoimmunotherapy: a multi-center study. npj Precis Onc. 2026 Mar 31. doi:10.1038/s41698-026-01388-z.

[3] She Y, He B, Wang F, Zhong Y, Wang T, Liu Z, et al. Deep learning for predicting major pathological response to neoadjuvant chemoimmunotherapy in non-small cell lung cancer: a multicentre study. eBioMedicine. 2022 Nov 14;86:104364.

[4] Ye G, Wu G, Qi Y, Li K, Wang M, Zhang C, et al. Non-invasive multimodal CT deep learning biomarker to predict pathological complete response of non-small cell lung cancer following neoadjuvant immunochemotherapy: a multicenter study. J ImmunoTher Cancer. 2024 Sep 3;12(9):e009348.

[5] Zheng J, Yan Z, Wang R, Xiao H, Chen Z, Ge X, et al. NeoPred: dual-phase CT AI forecasts pathologic response to neoadjuvant chemo-immunotherapy in NSCLC. J ImmunoTher Cancer. 2025 May 31;13(5):e011773.

[6] Ye G, Wei Z, Han C, Wu G, Wong C, Liang Y, et al. AI-derived longitudinal and multi-dimensional CT classifier for non-small cell lung cancer to optimize neoadjuvant chemoimmunotherapy decision: A multicentre retrospective study. eClinicalMedicine. 2025 Oct 7;89:103551.

**List of 520 genes included in the targeted sequencing panel.**

The panel covers full exons, partial exons, or introns of the following genes (listed in alphabetical order):

*ABL1, ABL2, ABRAXAS1, ACVR1, ACVR1B, AKT1, AKT2, AKT3, ALK, ALOX12B, AMER1, APC, AR, ARAF, ARFRP1, ARID1A, ARID1B, ARID2, ARID5B, ASXL1, ASXL2, ATM, ATR, ATRX, AURKA, AURKB, AXIN1, AXIN2, AXL, B2M, BAP1, BARD1, BBC3, BCL10, BCL2, BCL2L1, BCL2L11, BCL2L2, BCL6, BCOR, BCORL1, BIRC3, BLM, BMPR1A, BRAF, BRCA1, BRCA2, BRD4, BRD7, BRINP3, BRIP1, BTG1, BTG2, BTK, CALR, CARD11, CASP8, CBFB, CBL, CCND1, CCND2, CCND3, CCNE1, CD274, CD74, CD79A, CD79B, CDC73, CDH1, CDH18, CDK12, CDK4, CDK6, CDK8, CDKN1A, CDKN1B, CDKN1C, CDKN2A, CDKN2B, CDKN2C, CEBPA, CENPA, CHD1, CHD2, CHD4, CHEK1, CHEK2, CIC, CREBBP, CRKL, CRLF2, CSF1R, CSF3R, CSMD1, CSMD3, CTCF, CTLA4, CTNNA1, CTNNB1, CUL3, CUL4A, CXCR4, CYLD, CYP17A1, CYP2D6, DAXX, DCUN1D1, DDR1, DDR2, DICER1, DIS3, DNAJB1, DNMT1, DNMT3A, DNMT3B, DOT1L, DPYD, EED, EGFR, EIF1AX, EIF4E, EMSY, EP300, EPCAM, EPHA2, EPHA3, EPHA5, EPHA7, EPHB1, EPHB4, ERBB2, ERBB3, ERBB4, ERCC1, ERCC2, ERCC3, ERCC4, ERCC5, ERG, ERRFI1, ESR1, ETV4, ETV5, ETV6, EWSR1, EZH2, EZR, FANCA, FANCC, FANCD2, FANCE, FANCF, FANCG, FANCI, FANCL, FANCM, FAS, FAT1, FBXW7, FGF10, FGF12, FGF14, FGF19, FGF23, FGF3, FGF4, FGF6, FGF7, FGFR1, FGFR2, FGFR3, FGFR4, FH, FLCN, FLT1, FLT3, FLT4, FOXA1, FOXL2, FOXO1, FOXP1, FRS2, FUBP1, FYN, GABRA6, GATA1, GATA2, GATA3, GATA4, GATA6, GEN1, GID4, GLI1, GNA11, GNA13, GNAQ, GNAS, GPS2, GREM1, GRIN2A, GRM3, GSK3B, H3F3A, H3F3B, H3F3C, HDAC1, HDAC2, HGF, HIST1H1C, HIST1H2BD, HIST1H3A, HIST1H3B, HIST1H3C, HIST1H3D, HIST1H3E, HIST1H3G, HIST1H3H, HIST1H3I, HIST1H3J, HIST2H3D, HIST3H3, HLA-A, HLA-B, HLA-C, HNF1A, HOXB13, HRAS, HSD3B1, HSP90AA1, ICOSLG, ID3, IDH1, IDH2, IFNGR1, IGF1, IGF1R, IGF2, IKBKE, IKZF1, IL10, IL7R, INHA, INHBA, INPP4A, INPP4B, INSR, IRF2, IRF4, IRS1, IRS2, JAK1, JAK2, JAK3, JUN, KAT6A, KDM5A, KDM5C, KDM6A, KDR, KEAP1, KEL, KIT, KLF4, KLHL6, KMT2A, KMT2C, KMT2D, KRAS, LATS1, LATS2, LMO1, LRP1B, LTK, LYN, MAF, MAGI2, MALT1, MAP2K1, MAP2K2, MAP2K4, MAP3K1, MAP3K13, MAPK1, MAPK3, MAX, MCL1, MDC1, MDM2, MDM4, MED12, MEF2B, MEN1, MERTK, MET, MGA, MIR21, MITF, MKNK1, MLH1, MLH3, MPL, MRE11, MSH2, MSH3, MSH6, MST1, MST1R, MTAP, MTOR, MUTYH, MYC, MYCL, MYCN, MYD88, MYOD1, NAV3, NBN, NCOA3, NCOR1, NCOR2, NEGR1, NF1, NF2, NFE2L2, NFKBIA, NKX2-1, NKX3-1, NOTCH1, NOTCH2, NOTCH3, NOTCH4, NPM1, NRAS, NRG1, NSD1, NSD2, NSD3, NT5C2, NTHL1, NTRK1, NTRK2, NTRK3, NUP93, NUTM1, P2RY8, PAK1, PAK3, PAK5, PALB2, PARP1, PARP2, PARP3, PAX5, PBRM1, PCDH11X, PDCD1, PDCD1LG2, PDGFRA, PDGFRB, PDK1, PGR, PHOX2B, PIK3C2B, PIK3C2G, PIK3C3, PIK3CA, PIK3CB, PIK3CD, PIK3CG, PIK3R1, PIK3R2, PIK3R3, PIM1, PLCG2, PLK2, PMS1, PMS2, PNRC1, POLD1, POLE, PPARG, PPM1D, PPP2R1A, PPP2R2A, PPP6C, PRDM1, PREX2, PRKAR1A, PRKCI, PRKDC, PRKN, PTCH1, PTEN, PTPN11, PTPRD, PTPRO, PTPRS, PTPRT, QKI, RAB35, RAC1, RAD21, RAD50, RAD51, RAD51B, RAD51C, RAD51D, RAD52, RAD54L, RAF1, RARA, RASA1, RB1, RBM10, RECQL4, REL, RET, RHEB, RHOA, RICTOR, RIT1, RNF43, ROS1, RPA1, RPS6KA4, RPS6KB2, RPTOR, RSPO2, RUNX1, RUNX1T1, SDC4, SDHA, SDHAF2, SDHB, SDHC, SDHD, SETD2, SF3B1, SGK1, SH2B3, SH2D1A, SHQ1, SLC34A2, SLIT2, SLX4, SMAD2, SMAD3, SMAD4, SMARCA4, SMARCB1, SMARCD1, SMO, SNCAIP, SOCS1, SOX10, SOX17, SOX2, SOX9, SPEN, SPOP, SPTA1, SRC, SRSF2, STAG2, STAT3, STAT4, STAT5A, STAT5B, STK11, STK40, SUFU, SYK, TAF1, TBX3, TCF3, TCF7L2, TEK, TENT5C, TERC, TERT, TET1, TET2, TGFBR1, TGFBR2, TIPARP, TMEM127, TMPRSS2, TNFAIP3, TNFRSF14, TOP1, TOP2A, TP53, TP63, TRAF2, TRAF7, TRIM58, TRPC5, TSC1, TSC2, TSHR, TYRO3, U2AF1, UGT1A1, VEGFA, VEGFB, VHL, WISP3, WRN, WT1, XIAP, XPO1, XRCC2, XRCC3, YAP1, YES1, ZBTB16, ZBTB2, ZNF217, ZNF703, ZNRF3.*
